# Impact of Soybean Nodulation Phenotypes and Nitrogen Fertilizer Levels on the Rhizosphere Bacterial Community

**DOI:** 10.3389/fmicb.2020.00750

**Published:** 2020-05-12

**Authors:** Hao Wang, Chuntao Gu, Xiaofeng Liu, Chunwei Yang, Wenbin Li, Shaodong Wang

**Affiliations:** ^1^College of Life Science, Northeast Agricultural University, Harbin, China; ^2^Key Laboratory of Soybean Biology in Chinese Ministry of Education, Northeast Agricultural University, Harbin, China

**Keywords:** rhizosphere, bacterial community, diversity, soybean, nodulation phenotypes, nitrogen levels, super-nodulating

## Abstract

The effects of nodulation properties of legumes on the rhizosphere bacterial community are still not clear. To determine the effects of nodulation phenotypes on bacterial communities in the rhizosphere of soybean plants, we performed high-throughput sequencing of the 16S rRNA gene to estimate the rhizosphere bacterial community of three soybean lines with different nodulation phenotypes grown in soil supplied with different levels of N fertilizer. The results revealed that both the soybean nodulation phenotypes and the N levels affected the rhizosphere bacteria community, but the nodulation phenotypes contributed more than the N-supply. The diversity of bacteria was decreased in the rhizosphere of super-nodulating phenotype. The response of rhizosphere bacterial communities to the soil available nitrogen (AN) concentrations was different than the response with the three nodulation phenotypes of soybean which was more stable in the wild-type (Nod^+^) soybean samples than that in the mutant samples (Nod^–^ and Nod^++^). *Bradyrhizobium* in the rhizosphere was positively correlated with nodule number and negatively correlated to AN in the soil, while *Burkholderia* and *Dyella* were positively correlated with nodule biomass and nitrogenase activity. These results demonstrated that the nodulation phenotype of soybean affects the rhizosphere microbiome.

## Introduction

Soybean [*Glycine max* (L.) Merrill] is an important leguminous crop worldwide, and it can form a nitrogen-fixing symbiosis with some soil bacteria: so-called rhizobia. The SNF by soybean plants contributes 16.4 Tg of combined nitrogen (N) annually, accounting for 77% of the total N fixed by the legume crops (Herridge et al., 2008), which can meet 50–60% of the nitrogen demand of soybean plants during their life cycle (Salvagiotti et al., 2008). After harvesting, the crop residue of soybeans can also increase the nitrogen level of the soil (Kumar and Babalad, 2018). Therefore, improving the nodulation and nitrogen fixation capability of soybean is an important way to reduce the amount of nitrogen fertilizer usage and in turn to reduce the carbon emissions in fertilizer production.

The SNF could be promoted by the appropriate supply of nitrogen in its early growth stage (Marschner, 1995), but was inhibited by excess nitrogen supplementation (Zahran, 1999) through reducing the nodule numbers and nitrogenase activity. Thus, we should take into account of the nitrogen level in the soil for studying the nodulation of soybean. In wild-type (*nod*^+^) soybean, the number of nodules per plant is regulated by sophisticated machinery, so-called AON (Reid et al., 2011), and GmNARK has been identified as the key gene controlling the AON (Searle et al., 2003). The deficiency of AON could make the soybean plants as super-nodulating mutants (Akao and Kouchi, 1992), while non-nodulating soybean mutants were also obtained (Mathews et al., 1987).

The rhizosphere is the transit region between the surface of plant roots and the bulk soil in which the physicochemical features are strongly affected by the growth, respiration, and nutrient exchange of roots. Therefore, the microbial abundance, diversity, and activity in the rhizosphere are different from those in the vicinity of bulk soil and endosphere of the root (Xiao et al., 2017a, 2019). Numerous studies have demonstrated that plant root exudates mediate the interactions between plant roots and the microbial communities in the rhizosphere; for example, under N-limiting conditions, legumes secrete more flavones and flavonols to attract and initiate a symbiosis with rhizobia (Huang et al., 2014). Considering their SNF trait, the interactions among the microbes associated with the roots of leguminous plants are more complex than those in the rhizosphere of other plants. The differences in the microbiome in distinct rhizocompartments (nodule endophytes, root endophytes, rhizosphere, and root zone) of soybean and alfalfa have been revealed by high-throughput sequencing (Xiao et al., 2017a). Meanwhile, the effects of soil type on rhizomicrobiome of *Phaseolus vulgaris* estimated with a double pot system revealed that the bacterial community in rhizosphere is regulated by long-distance plant signaling (Xiao et al., 2019). These previous studies demonstrated that the root nodules are restricted microhabitats for both the rhizobia and the other bacteria.

Studies on the microbiota of soybean plants with different nodulation phenotypes have revealed that the stem- and leaf-associated bacterial communities were affected by the nodulation phenotypes and nitrogen fertilization levels (Ikeda et al., 2010, 2011). In addition, a ribosomal intergenic transcribed spacer analysis (RISA) described that the microbial communities (bacteria and fungi) associated with stems and roots varied with the different nodulation phenotypes of the soybean plants (Ikeda et al., 2008). Since the rhizosphere microbes can improve plant health and growth via different mechanisms (Huang et al., 2014), it has great value to learn the interactions between the rhizosphere bacteria and the soybean genotypes. Several studies have been performed on the diversity and community shifting in relation to the cultivars and growth stages of soybean, as well as to the soil types (Xu et al., 2009; Xiao et al., 2017b). However, in the previous studies, the effects of nodulation genotypes on the soybean rhizosphere microbial communities were not clearly described.

Considering the deficiency of information about the effects of nodulation genotypes on the rhizosphere microbes, we performed the present study to evaluate the impacts of soybean nodulation phenotypes and N fertilizer levels on the rhizosphere bacterial community, using the Illumina MiSeq platform.

## Materials and Methods

### Plant Materials, Soil, and Pot Culture Experimental Designs

The soybean lines used in this study were the spring cultivar Heihe 38 (wild-type nodulating cultivar; Nod^+^), the non-nodulating summer cultivar En 1282 (derived from non-nodulating mutant Enrei; Nod^–^) (Francisco and Akao, 1993), and the super-nodulating spring cultivar Dongfu 4 (a hybrid descendant derived from male parent super-nodulating mutant ZX 4 × female parent Heihe 38; Nod^++^). Some growth features of the three cultivars are presented in Supplementary Table S1.

The soil used in this study was sampled from 0 to 20 cm in a degraded wetland (typical field for local corn-soybean rotation) near the city of Harbin, China (GPS location: N45, E126), where corn was cultivated previously without the addition of N fertilizer. The soil was a sandy loam with the following characteristics: pH 7.65 ± 0.09, available N content 28.67 ± 1.07 mg/kg, P_2_O_5_ 47.3 ± 0.9 mg/kg, K_2_O 116.9 ± 1.9 mg/kg, Electrical Conductivity (EC) 0.050 ± 0.002 ds/m, salinity 0.002 ± 0%, and total organic carbon 3.69 ± 0.22 g/kg. The WHC of the soil was 32.6%, as determined by the gravity method. The soil physicochemical characteristics were determined using standard methods (Carter et al., 2007) in triplicate. To obtain the samples, soybean seeds were planted on May 15, 2018, in plastic pots (diameter, 11 cm; height, 40 cm) (patent No. ZL 2015 2 0193626.9), filled with 3.5 kg soil per pot with a moisture of 60% WHC. The plants were grown in a greenhouse with natural day/night cycling, and water was supplied from the bottom to maintain the moisture when necessary.

The experimental designation was four levels of N as urea (N0, 0 N fertilizer; N1, 50 mg N/kg soil; N2, 100 mg N/kg soil; and N3, 150 mg N/kg soil), combined with P as calcium superphosphate (450 mg/kg soil) and K as potassium sulfate (150 mg/kg soil), all in analytical grade. Six seeds were planted in each pot, but only one seedling remained after sprouting by thinning out the excess seedlings. The mixed fertilizers were dressed around the seedling root, following by watering. Six repeats were set for each treatment, covering a total of 72 pots (plants). The pots corresponding to different treatments were randomly arranged in the greenhouse.

### Sampling of Plants, Nodules, and Soils

Soil and plant samples were obtained at the full-bloom stage, for which the pots were split without damage to the soybean roots. After removing the roots, soils in pots of each treatment were mixed and air-dried for available N determination in triplicate with the standard method (Carter et al., 2007). For the collection of rhizosphere soil, three plants from each treatment were gently shaken to eliminate the excess root-attached soil particles and the soil adhering to the roots was brushed off with soft toothbrush. The rhizosphere soil samples were then stored at −80°C until use.

### Nodulation Characterization

Nitrogenase activity of the plants at full bloom stage was measured for the entire roots with nodules by the ARA according to Hardy et al. (1973) and Xia et al. (2017). Briefly, the intact roots of three plants from each treatment were cut at the cotyledonary node, washed, blotted for drying without detaching the nodules, and then placed separately in a 500-mL amber glass wide-mouth bottle fitted with a rubber stopper. 50 mL of air was replaced in each bottle with an equal volume of acetylene gas (at a concentration of 99.9%). After 2 h of incubation at room temperature, gas sample (5 mL) was transferred with a syringe to a 5-mL head space bottle (pre-evacuated). A GC 7900 gas chromatograph (Shanghai Techcomp Scientific Instrument Co., Ltd., China) was used for detecting the ethylene. Acetylene reduction activity (ARA) was expressed in the μmole of ethylene formed per plant per hour. The data were given as statistical analysis with the SPSS Statistics V 20.0 (SPSS: IBM Corp., United States) software.

### Biomass of Plants

Plant height was measured for each treatment at the sampling time. After ARA, the nodule number and fresh nodule weight were counted for each plant. Then, all the plant samples were dried at 60°C for 48 h to determine the dry weights of the aboveground part and the root. Chlorophyll content of the last but one ternate compound leaf was measured just before the plant was sampled by the portable chlorophyll detector (CCM-200, OPTI-Sciences, United States). All the data were obtained in triplicate for the subsequent statistic analysis.

### DNA Extraction and PCR Amplification

For microbial diversity analysis, metagenomic DNA was extracted from 0.25 g of the rhizosphere soil sample using the PowerSoil DNA Isolation kit (MO BIO, Carlsbad, CA, United States) according to the manufacturer’s protocol. The V4–V5 region of the bacterial 16S rRNA gene (about 400 bp) was amplified by PCR (95°C for 2 min, followed by 25 cycles at 95°C for 30 s, 55°C for 30 s, and 72°C for 30 s, with a final extension at 72°C for 5 min) with primers 515F (5′-barcode-GTG CCA GCM GCC GCG G)-3′ and 907R (5′-CCG TCA ATT CMT TTR AGT TT-3′). The barcode was an eight-base sequence, unique to each sample. PCR reactions were performed in triplicate in 20-μL mixture containing 4 μL of 5 × FastPfu Buffer, 2 μL of 2.5 mM dNTPs, 0.8 μL of each primer (5 μM), 0.4 μL of FastPfu Polymerase, and 10 ng of template DNA. Amplicons were extracted from 2% (w/v) agarose gels after electrophoresis and purified using the AxyPrep DNA Gel Extraction Kit (Axygen Biosciences, Union City, CA, United States) according to the manufacturer’s instructions and then quantified using the QuantiFluor^TM^-ST (Promega, United States).

### Library Construction and Sequencing

Purified PCR products were quantified by Qubit^®^3.0 (Life Invitrogen), and every 24 amplicons with different barcodes were mixed equally. The pooled DNA products were used to construct an Illumina Pair-End library following the Illumina genomic DNA library preparation procedure. Then, the amplicon library was paired-end sequenced (2 × 250) on an Illumina MiSeq platform (Shanghai BIOZERON Co., Ltd.), according to the standard protocols. The raw reads were deposited into the NCBI Sequence Read Archive (SRA) database under the accession number of SRP151632.

### Processing of Sequencing Data

Raw FASTQ files were demultiplexed and quality-filtered using QIIME (version 1.17) with the following criteria: (i) The 250-bp reads were truncated at any site receiving an average quality score of <20 over a 10-bp sliding window, discarding the truncated reads that were shorter than 50 bp. (ii) Exact barcode matching, 2-nucleotide mismatches in the primer matching, and reads containing ambiguous characters were removed. (iii) Only sequences with overlaps longer than 10 bp were assembled according to their overlap sequence. Reads that could not be assembled were discarded.

Operational taxonomic units (OTUs) were defined with a 97% similarity cutoff using UPARSE (version 7.1^1^), and chimeric sequences were identified and removed using UCHIME. We combined the three replicates into one sample by summing up the values of OTUs in the three replicates. The phylogenetic affiliation of each 16S rRNA gene sequence was analyzed by RDP Classifier^2^ against the SILVA (SSU119) 16S rRNA database using a confidence threshold of 70% (Amato et al., 2013).

### Alpha- and Beta-Diversity Analyses

Based on the results of OTU cluster analysis, the Alpha-diversity of the 12 samples was estimated by calculating the indices Chao, ACE, Shannon, and Simpson, while the sequencing coverage was also calculated for each treatment (Bai et al., 2015). Sequences in each OTU ranged from large to small according to the OTU richness, and the Rank-abundance curves were drawn with the relative abundances of each OTU ranked against the OTU ranks.

Principal coordinate analysis (PCoA) was performed with QIIME program to examine dissimilarities in the community composition of the samples by plotting the 12 samples in (12-1)-dimensional space, and the samples were grouped based on unweighted and weighted UniFrac distance metrics. The OTUs defined in this study were further compared with the defined bacteria to determine their species affiliation by blasting in the NCBI database, and the sequence similarity of 97% was used as the threshold of species.

### Redundancy Analysis (RDA)

Detrended correspondence analysis (DCA) showed that the largest axis length was 1.33 and 1.42 at the genus level. Consequently, RDA was selected, and the significance of nodulation characteristics, AN, and plant biomass factors were tested with Monte Carlo permutations (permu = 999). The analyses of RDA were conducted in *R* for statistical computing (R Development Core Team, 2013), using the vegan package (Oksanen et al., 2013).

## Results

### Nodulation and Growth Characterization of Soybean Cultivars

As expected, no nodule was observed on the roots of En 1282 (Nod^–^) plants in any treatments, while 129 and 900 nodules per plant on average were counted on the roots of Heihe 38 (Nod^+^) and Dongfu 4 (Nod^++^) plants in N0 treatments (Table 1), which fitted the symbiotic characters for each soybean cultivar. The results in Table 1 showed that the nodule numbers, nodule mass, and ARA of Dongfu 4 plants were significantly higher than those of the Heihe 38 plants at all four N levels. The nodule numbers of Dongfu 4 were decreased as the N levels increased from the N0 to N3 (no significant difference between N2 and N3) treatments, while no significant change was observed in the nodule numbers of the Heihe 38 plants at the four N levels. The nodule mass of the Heihe 38 plants was significantly decreased in the N2/N3 treatments compared with the N0/N1 treatments, but this value of Dongfu 4 was significantly increased in the N1 treatment relative to the N0 treatment; then, the nodule mass of Dongfu 4 gradually decreased in the N2 and N3 treatments. For the ARA, the Dongfu 4 plants presented significantly greater values in the N1 and N2 treatments than those in the N0 and N3 treatments, while no significant difference was observed in the Heihe 38 plants at all the four N levels.

**TABLE 1:** Effects of Nitrogen levels on the nodulation characteristics of different soybean cultivars.

Sample code	Treatment		Nodule no. (plant^–1^)	Nodule mass (g plant^–1^)	Ethylene (μmol/plant/h)	Available nitrogen (mg/Kg)	Chlorophyll content (SPAD)	Height (cm)	Dry weight of upground (g/Plant)	Dry weight of root (g/Plant)
	Cultivar	N-supp. mg/kg								
H-N0	Heihe 38	0	129.67 ± 27.43a	6.50 ± 1.01b	92.37 ± 23.42a	53.02 ± 2.22a	18.81 ± 2.38cd	27.82 ± 2.87bc	9.24 ± 1.18ab	5.13 ± 0.70abc
H-N1		50	255.33 ± 21.55a	6.93 ± 0.76b	125.59 ± 24.34a	56.49 ± 2.03abc	16.67 ± 0.84bc	34.42 ± 4.64d	12.25 ± 3.84b	5.91 ± 1.15c
H-N2		100	207.00 ± 14.18a	4.03 ± 0.61a	131.13 ± 32.81ab	62.57 ± 3.32d	21.38 ± 2.76de	30.17 ± 4.40cd	14.67 ± 3.68b	6.42 ± 0.78c
H-N3		150	123.67 ± 46.48a	3.00 ± 0.90a	75.53 ± 44.37a	70.52 ± 2.49e	22.51 ± 5.16de	31.18 ± 5.54cd	12.29 ± 1.49b	5.28 ± 1.96bc

D-N0	Dongfu 4	0	900.67 ± 219.00d	13.53 ± 1.76c	210.21 ± 61.08c	52.21 ± 5.57a	25.31 ± 2.62ef	22.33 ± 3.85a	6.16 ± 0.67a	2.51 ± 0.15a
D-N1		50	684.00 ± 154.28c	19.32 ± 1.55e	351.85 ± 34.32d	60.57 ± 2.67cd	27.39 ± 1.55f	28.65 ± 3.95c	10.72 ± 2.1ab	2.94 ± 0.24ab
D-N2		100	490.00 ± 43.59b	17.00 ± 1.61d	298.84 ± 39.32d	76.03 ± 3.75f	27.47 ± 4.18f	27.08 ± 3.00abc	8.70 ± 1.40ab	3.11 ± 0.39ab
D-N3		150	465.67 ± 55.82b	13.17 ± 2.10c	196.70 ± 30.76bc	58.40 ± 2.92bcd	26.67 ± 4.48f	31.55 ± 2.35cd	11.29 ± 0.98ab	3.11 ± 0.65ab

E-N0	En 1282	0				60.00 ± 0.33bcd	5.57 ± 1.15a	23.02 ± 3.11ab	12.37 ± 0.56b	6.02 ± 0.57c
E-N1		50				55.27 ± 1.36ab	7.44 ± 0.70a	26.60 ± 57.07abc	27.77 ± 3.51c	10.32 ± 2.02e
E-N2		100	Without nodule			60.88 ± 1.06cd	7.23 ± 1.32a	28.78 ± 4.50c	47.75 ± 7.16d	7.68 ± 2.23cd
E-N3		150				61.31 ± 1.26cd	13.92 ± 4.18b	34.30 ± 3.79d	32.40 ± 4.35c	9.74 ± 3.00de

**Data are mean ± standard variation (n = 3). Different lowercase letters in the same column show significant differences between treatments (P < 0.05).* “H, D, E” represent the soybean cultivars “Heihe 38, Dongfu 4 and En1282” and “N0, N1, N2, N3” represent the four levels of N as urea, “0 N fertilizer; 50 mg N/kg soil; 100 mg N/kg soil; 150 mg N/kg soil”.*

In this study, the chlorophyll contents of leaves presented in the order of Dongfu 4 > Heihe 38 > En 1282, while it was constant in Dongfu 4 and Heihe 38 at all the four N levels, but significantly increased in En 1282 at the highest N level. For plant height, both the nodulation cultivars presented a positive response to the N1 level and a supplement of more N fertilizer did not cause more growth, while the Nod^–^ cultivar En 1282 presented the positive response only for the highest N level (N3). For the dry weight of aerial parts, the three cultivars presented the order of En 1282 > Heihe 38 > Dongfu 4. While the dry weight was constant for the two nodulating cultivars despite the N levels, it was significantly increased for En 1282 from N0 through N2 levels and decreased at N3 level. For root biomass, the situation was similar to that of aerial parts (Table 1).

### Bacterial Community

In the high-throughput DNA sequencing, 1,115,186 valid sequences with average lengths of 396 bp were obtained from the 12 samples, after filtering the raw reads (Table 2). The optimized sequences were divided into 3,310 OTUs at 97% sequence identity after cluster analysis. The coverage for all the samples was >99%, implying that almost all of the OTUs were detected. The Alpha-diversity in the 12 samples (Table 2) showed that the samples of En 1282 at different nitrogen levels had the highest diversity and that of Dongfu 4 had the lowest diversity. Within the single cultivars, samples of Dongfu 4 and En 1282 showed higher community diversity at the N1 level than at the other three N levels, while the samples of Heihe 38 exhibited a decreased diversity as the N level increased.

**TABLE 2:** Comparison of the observed OTUs and Estimators of bacterial communities in the rhizosphere of three nodulation phenotypes soybean at different N levels.

Sample ID*	Reads	OTU	Ace	Chao	Coverage	Shannon	Simpson
D-N0	86014	2037	2596 ± 93	2593 ± 117	0.99	3.32 ± 0.02	0.23 ± 0.00
D-N1	83282	2404	2804 ± 70	2827 ± 93	0.99	4.27 ± 0.02	0.11 ± 0.00
D-N2	78574	2142	2708 ± 92	2695 ± 115	0.99	3.47 ± 0.02	0.22 ± 0.00
D-N3	92995	2197	2658 ± 78	2662 ± 101	0.99	3.42 ± 0.02	0.21 ± 0.00

E-N0	91206	2767	3044 ± 52	3026 ± 63	0.99	5.02 ± 0.02	0.09 ± 0.00
E-N1	72048	2693	3037 ± 62	3071 ± 86	0.99	6.00 ± 0.02	0.01 ± 0.00
E-N2	72318	2422	2851 ± 74	2880 ± 100	0.99	5.03 ± 0.02	0.05 ± 0.00
E-N3	80078	2358	2805 ± 77	2833 ± 103	0.99	5.07 ± 0.01	0.04 ± 0.00

H-N0	89451	2786	3086 ± 56	3113 ± 77	0.99	5.55 ± 0.02	0.03 ± 0.00
H-N1	74059	2514	2938 ± 72	2943 ± 93	0.99	4.97 ± 0.02	0.05 ± 0.00
H-N2	68345	2195	2717 ± 86	2710 ± 108	0.99	4.21 ± 0.02	0.10 ± 0.00
H-N3	68591	1983	2683 ± 111	2696 ± 144	0.99	3.21 ± 0.03	0.22 ± 0.00

*See footnote of Table 1 for the corresponding treatments.

The rarefaction curves (available as Supplementary Figure S1) obtained using the randomly selected sequences trended to be flat when the number of sequences was >40,000, indicating that most OTUs have been recovered and that the sizes of the sequencing data were reasonable for our analysis. The rank-abundance curve (Supplementary Figure S2) obtained in this study demonstrated that the OTUs with relative abundance values as low as 0.001 were found in each sample and that most of the OTUs were recovered in this study. The community composition at the genus level is summarized in Supplementary Table S2.

### Influence of Cultivar and N Supply on Bacterial Diversity

**FIGURE 1:**
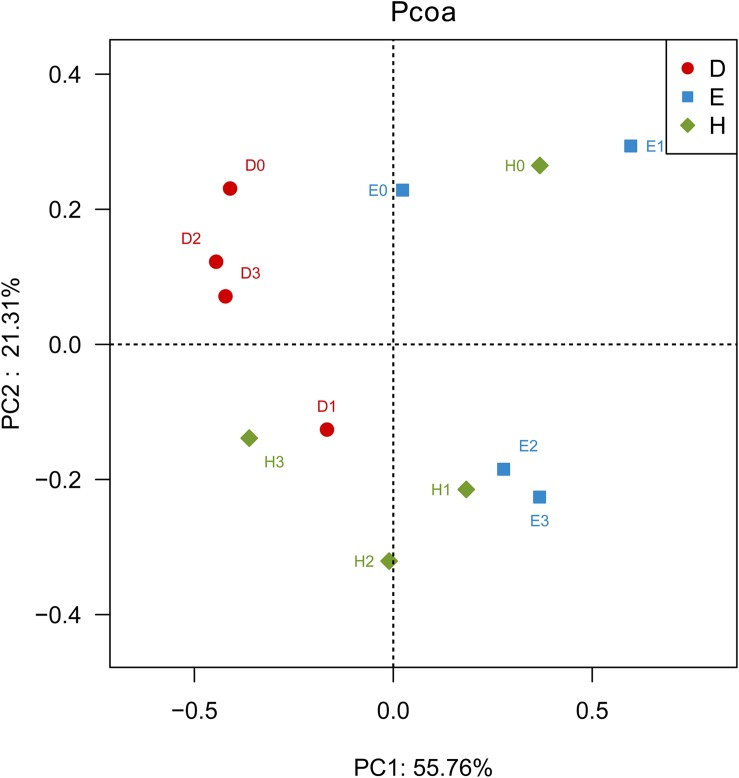
Principal coordinate analysis (PCoA) based on the Bray–Curtis distance metrics of bacterial communities in the rhizosphere of soybeans with different nodulation phenotypes at different nitrogen levels (*n* = 36). *D* represents the rhizospheric soils of the line Dongfu 4; *E* represents the rhizospheric soils of the line En 1282; *H* represents the rhizospheric soils of the cultivar Heihe 38. Arabic numerals 0, 1, 2, and 3 represent the Nitrogen levels.

The PCoA based on the Bray-Curtis distance metrics of OTU distribution generated two principal cooradinate (PCs), which collectively explained nearly 77% of the variation among the samples (Figure 1). In the direction of PC1, the communities in the rhizosphere of Dongfu 4 were separated from those in the rhizosphere of En 1282, while the communities in Heihe 38 rhizosphere were distributed at both sides of the *Y*-axis. These results indicated that the nodulation phenotypes of the soybeans might affect the community composition of rhizosphere bacteria, as they accounted for the largest source of variation (55.76%). In the direction of PC2, H0 was distinct from H1, H2, and H3; D1 was separated from D0, D2, and D3; and E0 and E1 were different from E2 and E3. These results revealed that the N level might be the second factor used to explain the 21.31% variation of the community composition of rhizosphere bacteria.

### Community Similarity and Differences Between the Different Samples

**FIGURE 2:**
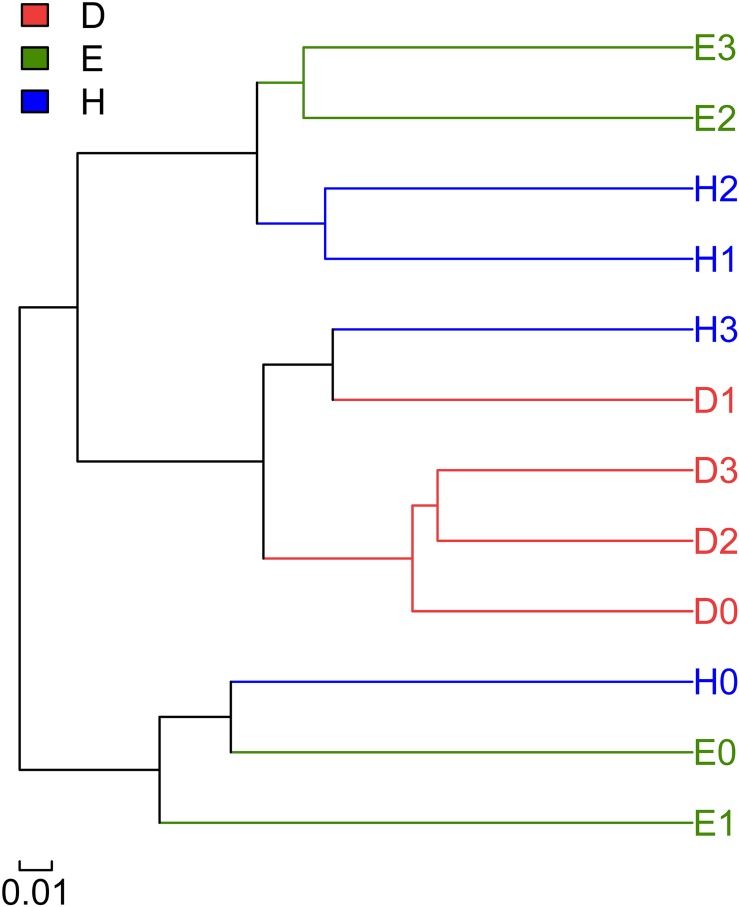
Multiple sample similarity tree showing the relationships among the rhizosphere microbial communities in the three soybean lines and the four N levels. *D*: rhizospheric soil of line Dongfu 4; *E*: rhizospheric soil of line En 1282; *H*: rhizospheric soil of cultivar Heihe 38. The numbers 0, 1, 2, and 3 represent the nitrogen levels.

A hierarchical clustering tree was constructed to describe and compare the similarities of multiple samples (Figure 2). Based on the similarities between the community compositions, the 12 soil samples were divided into three groups. The first group included the bacterial communities of the Heihe 38 rhizosphere at the N3 level and the bacterial communities of the Dongfu rhizosphere at all the four N levels, implying that the bacterial community compositions of the Dongfu rhizosphere were not considerably affected by the N levels. The second group covered the communities of Heihe 38 rhizosphere at N0 level and the samples of En1282 at N0 and N1 levels. The third group was composed of bacterial communities of Heihe 38 rhizosphere at N1 and N2 levels together with the samples of En1282 at N2 and N3 levels.

### Response of Bacterial Community to the Nodulation Characteristics, Available N, and Plant Biomass

The available N in soil and plant biomass observed in different treatments are shown in Table 1. In general, the available N (AN) in soil (ranging between 52 and 76 mg/kg) was increased across all the treatments after culturing soybean with or without N supply. As to the cultivar, the AN in Heihe 38 cultured soil was similar at N0 and N1 levels and increased at N2 and N3 levels; in Dongfu 4 cultured soil the AN was significantly increased in all the N supplied treatments; and in En 1282 cultured soil, the AN contents are almost the same, despite the doses of N supplement.

**FIGURE 3:**
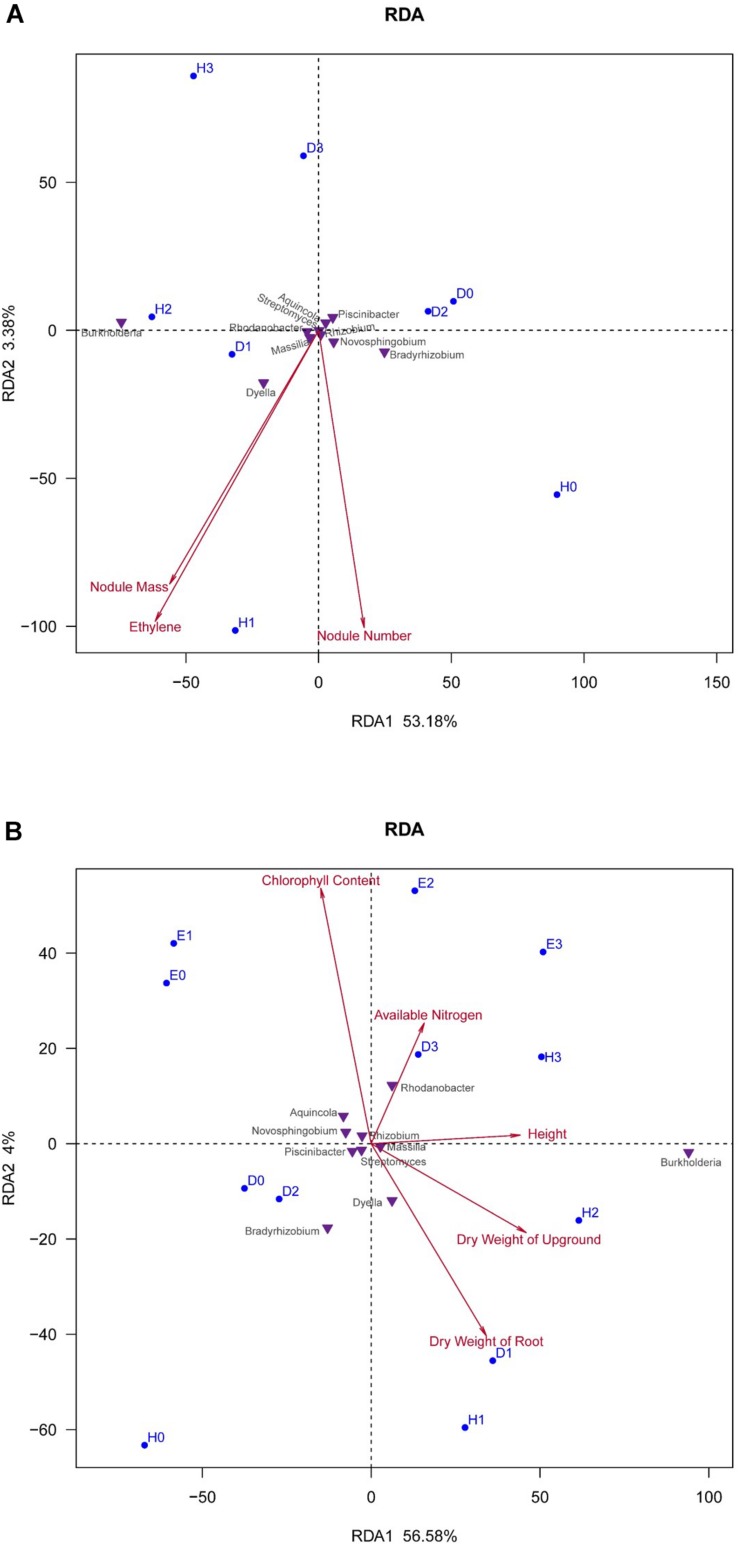
Redundancy analysis (RDA) plots showing the response of soybean rhizosphere bacterial communities to the nodulation characteristics, available nitrogen (AN) and plant biomass factors. **(A)** Relationship of rhizosphere bacterial community composition (at the genus level) with the nodulation characteristics in the treatments of super-nodulating and normal-nodulating soybean cultivars supplied with different nitrogen fertilizer. **(B)** Relationship of rhizosphere bacterial community composition (at the genus level) with the soil AN and plant biomass factors in the treatments of super-nodulating, normal-nodulating, and non-nodulating soybean cultivars.

The response of soybean rhizosphere bacterial community to the nodulation characteristics, AN in soil, and plant biomass factors were expressed by RDA plots in Figure 3. It could be observed that the nodulation characteristics (nodule biomass, nodule number, and nitrogenase activity) contributed a total of 56.56% (RDA1 + RDA2) for the variance in the bacterial communities (Figure 3A); while the soil AN and plant biomass contributed 60.58% (RDA1 + RDA2) of the bacterial community variation (Figure 3B). The sum of contributions in Figures 3A,B greater than 100% (117.14%) might be explained by the interactions between the nodulation characters and the plant biomass factors. In general, the correlations between the nodulation characters of the soybean cultivars and the bacterial communities were demonstrated by their distances of foot points to arrows in the plot (Figure 3A). The nodule mass and nitrogenase activity (ethylene production) presented a very similar correlation to the bacterial communities. The H-N1 (wild-type Heihe 38–50 mg N) community showed the highest and positive correlation with all the three nodulation characters, followed by that of H-N2, D-N1, and H-N0 samples; while the rhizosphere bacterial communities in D-N2, D-N0, D-N3, and H-N3 samples were negatively correlated to the nodulation characters (Figure 3A). As for the bacterial genera, *Dyella* and *Bradyrhizobium* in the rhizosphere were positively correlated with the nodule number, while *Burkholderia* and *Dyella* were positively correlated to the nodule mass and nitrogenase activity (Figure 3A).

In Figure 3B, it seemed that the rhizosphere bacterial communities in all the treatments were highly correlated by the chlorophyll content and plant biomass (shoot and root weight). The response of bacterial communities to the soil AN was enhanced with the increase of N levels for the wild-type soybean (Heihe 38) samples. The rhizosphere bacterial communities in super-nodulating soybean (Dongfu 4) samples D-N0, D-N1, and D-N2 had a similar correlation to the AN in soil, and D-N3 has a high correlation to the AN. The rhizosphere bacterial communities of non-nodulating soybean samples E-N0 and E-N1 have a higher correlation to soil AN than those of E-N2 and E-N3. The rhizosphere communities of bacteria of non-nodulating AON mutant En 1282 had a higher correlation with the chlorophyll content than those of the nodulating cultivars. Moreover, at the genus level, *Bradyrhizobium* and *Dyella* were negatively correlated with the AN, while *Rhodanobacter* had the highest positive correlation with the AN.

## Discussion

As one of the most important legume crops, many studies related to the SNF of soybeans have been performed, assessing the amounts of nitrogen required for promoting or inhibiting the nodulation (Marschner, 1995; Zahran, 1999), the diversity and effectiveness of its symbionts (Chen et al., 2002; Yan et al., 2017), and the genes involved in nodulation/nitrogen fixation in both soybean plants (Searle et al., 2003; Ikeda et al., 2008; Lim et al., 2010) and rhizobia (Shamsel, 2013). However, to the best of our knowledge, the effects of nodulation characters on soybean rhizosphere microbes have rarely been studied. As a part of root system for N_2_-fixation, the presence and number of nodules on the roots might affect soybean rhizosphere bacteria.

In the present study, we used soybean cultivars with normal, absent, and enhanced nodulation abilities to reveal the effects of nodulation phenotypes and N fertilization on symbiosis formation and rhizosphere bacteria composition. To eliminate the impact of soil type on the microbiomes in the rhizosphere (Liu et al., 2019), only one soil was used in this study. The results indicated that the non-nodulating AON mutant En 1282, super-nodulating AON mutant Dongfu 4, and wild-type nodulating cultivar Heihe 38 formed a set of models for investigating the effects of nodulation on the rhizosphere bacterial community. Indeed, the nodulation characters regulated the rhizosphere bacterial communities, since the Chao value and Shannon index at the treatments N0 (no N supply) demonstrated a tendency of Nod^+^ > Nod^–^ > Nod^++^ (Table 2). In other words, in the soil with a background N level of 28.67 mg/kg, the wild-type cultivar Heihe 38 harbored the most diverse rhizosphere bacteria, while the AON mutants (Nod^–^ and Nod^++^) decreased the diversity of rhizosphere bacteria. Previously, differences in rhizosphere microbiomes were observed among various cultivars (Wang et al., 2014), but all were nodulation wild types. In Ikeda et al. (2005), different compositions in root-associated microbiomes were revealed by a ribosomal intergenic spacer analysis (RISA), but no diversity index was reported for the microbiomes. So, our study was the first one to connect the diversity of rhizosphere microbiome with the nodulation genotypes. Since the diversity tendency was changed according to the nodulation phenotypes when the N level increased, we estimated that both the nodulation phenotype and the N levels regulated the diversity of rhizosphere bacteria, but the nodulation characteristics contributed more (56.56%) (Figure 3A) than the N levels under the conditions in this study (Figures 1, 2).

The effects of nodulation phenotype and N fertilizer levels on the rhizosphere bacteria were further revealed by the grouping of all the Dongfu 4 rhizosphere samples (D0 through D3) in the same cluster (Figures 1, 2). It seems that the bacterial community in the rhizosphere of the super-nodulating mutant (Dongfu 4) was not so sensitive to the soil nitrogen level, which might be related to the effects of nodulation traits on the rhizosphere bacteria, since it always presented a great number of nodules and high nitrogenase activity against different levels of N fertilizer.

It is well known that excessive N fertilization decreases the diversity of microbes in rhizosphere and bulk soils (Sun et al., 2019; Wang et al., 2019). In the present study, the decrease of the diversity of rhizosphere bacteria at the high dose of N fertilization (N2 and N3) compared with that in the N1 treatment for all the three cultivars (Table 2) demonstrated that only the excessive N fertilization decrease the diversity of rhizosphere bacteria, which might be through the selection pressure of high concentrated AN and alteration of soil pH on the microbes (Li et al., 2016). Also, some of the plant physiological features, such as the chlorophyll content and leaf area index, are regulated by the N level (Basal and Szabó, 2018), which could in turn affect the rhizosphere microbes via changing root exudates or signaling of the plant (Pfenning et al., 2009).

Previously, it has been reported that the nodulation and nitrogenase activity of soybean plants were stimulated by low-level N fertilization, and inhibited by high level (>50 mg-N/L) N fertilization (Xia et al., 2017). In general, similar results were observed in our present study (Table 1). However, the nitrogenase activity was more sensitive to the concentration of N fertilizer in Nod^++^ than in the Nod^+^ phenotypes, which were also found among other soybean cultivars (Abdel Wahab and Abd-Alla, 1995). Although the mechanism for the difference in sensitivity of nitrogenase to N-supply among the cultivars is not clear, King and Purcell (2005) described nodule ureides, nodule aspartate, and several amino acids (Asp, Gln, etc.) in leaves as the possible molecules for feedback inhibition of nitrogen fixation of soybean.

The similar bacterial community compositions in rhizospheres of the treatments D1 and H3 (Figures 1, 2) might imply that similar root exudates were produced by soybean plants in these two treatments, since the rhizosphere microbiome was strongly regulated by root exudates (Haichar et al., 2008; Subbarao et al., 2009; Huang et al., 2014; White et al., 2015; Szoboszlay et al., 2016) and compounds sloughed off root tips (Bulgarelli et al., 2013), which could be related to N fertilization, colonization of endophytic microbe, and N_2_ fixation of legume plant (Xie et al., 2019). To confirm this estimation, a comparative study on the root exudates is needed.

The greater similarities among the rhizosphere bacterial community compositions among the treatments of E0, E1, and H0, as well as among E2, E3, H1, and H2 (Figures 1, 2), implied that the Biological nitrogen fixation (BNF) by Heihe 38 nodules and N supply for En 1282 have similar effects on rhizosphere microbiomes. The mechanism of these effects needs further study, such as comparative analysis of root exudates of the soybean plants in the corresponding treatments. All these results suggested again that both the nodulation characteristics of the soybean plants and the nitrogen supply regulated the rhizosphere bacterial community, and their contribution varied depending on the cultivars of soybean plants.

In the RDA analysis, the *Bradyrhizobium* abundance in the rhizosphere was positively correlated with the nodule number, as *Bradyrhizobium* was the main microsymbionts of soybean in the region where the tested soil was taken (Yan et al., 2014, 2017). The positive correlation of *Burkholderia* with nodule mass and ethylene production demonstrated it to be beneficial for BNF, which was consistent with other reports that some PGPB *Burkholderia* strains could activate the energy production pathways of plants under both aerobic and microaerobic conditions, and in turn promote the BNF (Chanyarat et al., 2016). The positive correlation of *Dyella* with the nodule number/mass and ethylene production suggested it to be a possible PGPR, since *Dyella* isolated from the nodules of *Lespedeza* sp. could enhance the plant growth (Pitchai et al., 2010), probably by their production of indole acetic acid or 1-aminocyclopropane-1-carboxylate (ACC) deaminase (Correa-Galeote et al., 2018).

As an important biochemical parameter of plants, chlorophyll content has been used to estimate plant productivity and health status (Casa et al., 2015; Chen et al., 2017). However, the orders of Dongfu 4 > Heihe 38 > En 1282 for chlorophyll content and En 1282 > Heihe 38 > Dongfu 4 for the biomass of shoots and roots across all N treatments (Table 1) are somewhat surprising. We postulate that this counterintuitive situation might be related to the nodulation and BNF activities of the cultivars. Previously, it has been reported that the colonization and BNF of the endophytic bacteria could decrease the biomass accumulation of poplar and cedar plants, especially in the early growth stage (Anand and Chanway, 2013; Knoth et al., 2014). It was estimated that about 5.6–8.0 g of carbon were lost for fixing 1 g of N in the nodulation legumes (Phillips, 2003). So, we speculate that the greater BNF activity would require greater photosynthesis (more chlorophyll content) since BNF is an energy-consuming process. Therefore, the higher nodule number/biomass and higher ARA values in Dongfu 4 treatments than those in Heihe 38 treatments might explain why the chlorophyll contents in Dongfu 4 were the highest, but why its biomass was the lowest (Table 1). It is possible that in Dongfu 4 and Heihe 38, a remarkable proportion of carbohydrates produced by photosynthesis was used for N_2_ fixation.

Another point in this study is the fact that the amounts of AN in the soil samples at harvest are similar across all treatments, despite the significant differences in N supply at the beginning, which is similar to the results in previous report (Eickhout et al., 2006). In the N0 treatments, the increase of AN in the soil might from the BNF by rhizobia for the nodulating cultivars (Heihe 38 and Dongfu 4) and from BNF by other diazotrophic bacteria for the non-nodulating cultivar En 1282. Indeed, the abundances of diazotrophs (*Azoarcus*, *Azospira*, *Azospirillum*, *Azotobacter*, and *Azovibrio*) (Supplementary Table S2) were greater in the rhizosphere of En 1282 and Heihe 38 than that of Dongfu 4. In the other treatments, the supplied N might be removed by plant absorption, NO_3_^–^ leaching, denitrification, and NH_3_ volatilizing as reported in fields of other crops (Raun and Johnson, 1999; Eickhout et al., 2006). Also, high N supply could decrease the utilization efficiency of N fertilizer by crops (Raun and Johnson, 1999; Eickhout et al., 2006) and enhance the denitrification as high as 10 times (up to 0.3 to 1.0 kg N ha^–1^ day^–1^) in the pasture soil (Colbourn, 1992). Considering the fact that the biomass of soybean was not significantly increased by the addition of N fertilizer for two nodulation cultivars and, in addition, an increase of height was obtained at N1 level and the height was not further increased at N2 and N3 levels, demonstrating that the symbiotic BNF might completely fit the N nutrient requirement of soybean growth, excessive supply of N fertilizer was not necessary, as reported in other studies (Ju et al., 2009).

Conclusively, (1) both the nodulation characters and the N level affected the bacterial community in soybean rhizosphere, but the soybean nodulation phenotypes contributed more than the N-supply; (2) the diversity of bacteria was decreased in the rhizosphere of super-nodulating phenotype; (3) the responses of rhizosphere bacterial communities to the soil AN concentrations varied according to the nodulation phenotypes of soybean, which was more stable in the wild-type (Nod^+^) soybean samples than that in the mutant samples (Nod^–^ and Nod^++^); (4) *Bradyrhizobium* in the rhizosphere was positively correlated with nodule number and negatively correlated to AN in the soil, while *Burkholderia* and *Dyella* were positively correlated with nodule biomass and nitrogenase activity. These results demonstrated that the nodulation phenotype of soybean affects the rhizosphere microbiome.

## Data Availability Statement

The datasets generated for this study can be found in the NCBI Sequence Read Archive (SRA) database (Accession Number: SRP151632).

## Author Contributions

HW was the first and corresponding author of this article, who was responsible for the manuscript writing and organizing research. CG was mainly responsible for bioinformatics analyzing. XL and CY were responsible for sample collection and processing. WL was responsible for linguistic modification. SW was responsible for field management.

## Conflict of Interest

The authors declare that the research was conducted in the absence of any commercial or financial relationships that could be construed as a potential conflict of interest.

## Footnotes

1 http://drive5.com/uparse/

2 http://rdp.cme.msu.edu/

## Abbreviations

AN: available nitrogen
AON: autoregulation of nodulation
ARA: acetylene reduction assay
DCA: detrended correspondence analysis
GmNARK: *Glycine max* nodule autoregulation receptor kinase
N: nitrogen
OTUs: operational taxonomic units
PGPB: plant growth-promoting bacteria
RDA: redundancy analysis
SNF: symbiotic nitrogen fixation
WHC: water holding capacity.
