# Supplementary material for: Impact of Soybean Nodulation Phenotypes and Nitrogen Fertilizer Levels on the Rhizosphere Bacterial Community
Source: Front Microbiol. 2020 May 12;11:750. doi: 10.3389/fmicb.2020.00750 (PMC7247815; doi:10.3389/fmicb.2020.00750)
Supplement: Supplementary file 1 [file Data_Sheet_1.docx]

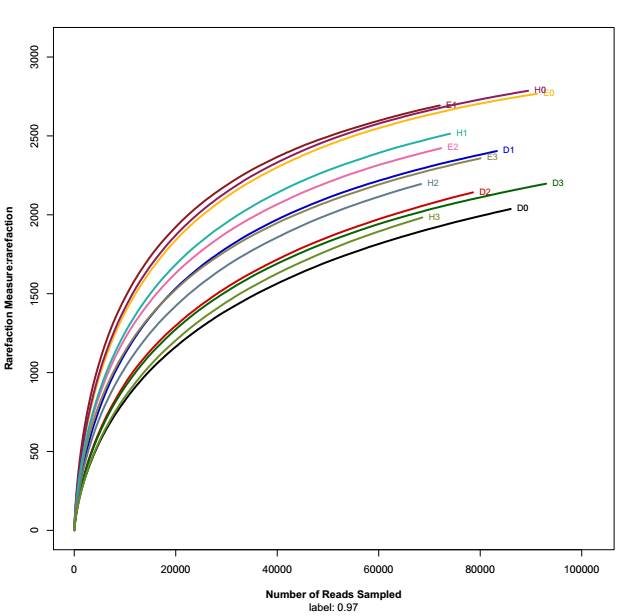


Supplementary Fig. S1. Rarefaction curves of OTUs clustered at <97% sequence identity for the three soybean cultivars’ rhizoshpere soil sampled from different N levels.


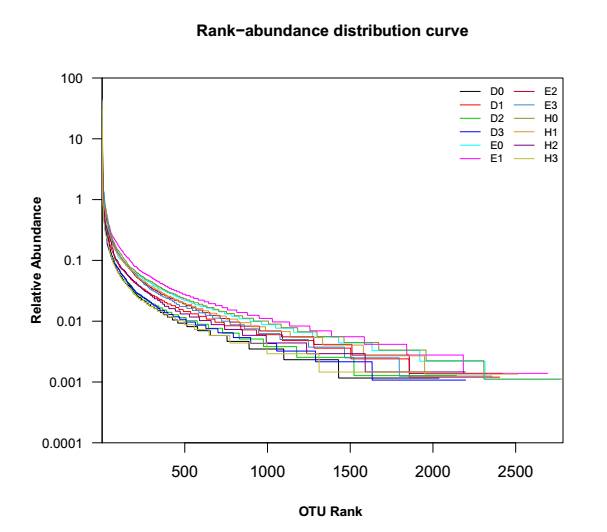


Supplementary Fig. S2. OUT Rank-abundance curves for the OTUs in the 12 soybean rhizosphere soil samples.
