# Supplementary material for: Impact of Soybean Nodulation Phenotypes and Nitrogen Fertilizer Levels on the Rhizosphere Bacterial Community
Source: Front Microbiol. 2020 May 12;11:750. doi: 10.3389/fmicb.2020.00750 (PMC7247815; doi:10.3389/fmicb.2020.00750)
Supplement: Supplementary file 2 [file Data_Sheet_2.docx]

**Supplementary Table S1**. Growth characters of the three soybean cultivars used in this study

| **Cultivar** | **Sowing season** | **Growth period (d)** | **Average Height (cm)** | **Photoperiod sensitivity** | **Average Yield (Kg·ha^-1^)** |
| --- | --- | --- | --- | --- | --- |
| Dongfu 4 | Spring | 105-108 | 75 | No | 2400 |
| En 1282 | Summer | 130 | 100 | Yes | 2800 |
| Heihe 38 | Spring | 110 | 75 | No | 2600 |

**Supplementary Table S2.** The composition of the different rhizosphere bacterial communities at genus level in the twelve soil samples

| OTU ID | D0 | D1 | D2 | D3 | E0 | E1 | E2 | E3 | H0 | H1 | H2 | H3 |
| --- | --- | --- | --- | --- | --- | --- | --- | --- | --- | --- | --- | --- |
| 0319-6M6_norank | 22 | 32 | 25 | 30 | 49 | 25 | 24 | 29 | 41 | 30 | 26 | 25 |
| 08D2Z23_norank | 1 | 1 | 1 | 0 | 0 | 1 | 1 | 0 | 0 | 0 | 1 | 0 |
| 11-24_norank | 68 | 142 | 57 | 72 | 172 | 203 | 109 | 88 | 151 | 127 | 67 | 38 |
| 1921-3_norank | 1 | 0 | 0 | 2 | 0 | 0 | 0 | 2 | 0 | 0 | 1 | 1 |
| 1959-1_norank | 0 | 1 | 1 | 0 | 1 | 0 | 0 | 0 | 1 | 0 | 0 | 0 |
| 288-2_norank | 4 | 9 | 8 | 8 | 7 | 9 | 7 | 8 | 9 | 6 | 1 | 6 |
| 34P16_norank | 4 | 2 | 6 | 8 | 5 | 3 | 1 | 0 | 6 | 7 | 0 | 1 |
| 43F-1404R_norank | 2 | 1 | 2 | 0 | 6 | 2 | 1 | 1 | 5 | 1 | 1 | 0 |
| 480-2_norank | 32 | 63 | 74 | 82 | 186 | 110 | 76 | 101 | 108 | 66 | 43 | 62 |
| 7B-8_norank | 0 | 0 | 2 | 2 | 1 | 0 | 4 | 0 | 5 | 1 | 0 | 0 |
| A0839_norank | 14 | 18 | 5 | 17 | 49 | 30 | 21 | 18 | 30 | 39 | 16 | 12 |
| ABS-19_norank | 147 | 267 | 112 | 156 | 249 | 280 | 166 | 183 | 396 | 266 | 126 | 109 |
| AKIW1012_norank | 0 | 0 | 1 | 0 | 0 | 1 | 5 | 2 | 6 | 2 | 2 | 0 |
| AKIW659_norank | 2 | 0 | 2 | 1 | 4 | 3 | 4 | 0 | 3 | 3 | 1 | 1 |
| AKIW781_norank | 31 | 76 | 108 | 92 | 69 | 96 | 83 | 46 | 51 | 64 | 35 | 30 |
| AKYG1722_norank | 1 | 16 | 6 | 8 | 5 | 9 | 6 | 16 | 8 | 7 | 8 | 7 |
| AKYH478_norank | 15 | 27 | 21 | 21 | 73 | 74 | 29 | 25 | 47 | 59 | 8 | 17 |
| AKYH767_norank | 10 | 26 | 9 | 13 | 34 | 34 | 16 | 12 | 28 | 30 | 17 | 6 |
| AT-s3-28_norank | 2 | 2 | 1 | 0 | 4 | 2 | 0 | 1 | 1 | 3 | 2 | 2 |
| AT425-EubC11 terrestrial group_norank | 20 | 33 | 28 | 38 | 75 | 80 | 65 | 86 | 103 | 85 | 29 | 25 |
| Acetobacteraceae_Unclassified | 4 | 5 | 4 | 5 | 10 | 14 | 17 | 9 | 20 | 11 | 5 | 6 |
| Acetobacteraceae_uncultured | 10 | 7 | 10 | 16 | 36 | 18 | 24 | 20 | 29 | 16 | 13 | 14 |
| Acidaminobacter | 0 | 0 | 0 | 0 | 0 | 0 | 0 | 0 | 9 | 0 | 0 | 0 |
| Acidibacter | 213 | 153 | 138 | 149 | 386 | 221 | 168 | 106 | 360 | 159 | 149 | 92 |
| Acidiferrobacter | 5 | 13 | 6 | 6 | 14 | 18 | 1 | 2 | 12 | 13 | 2 | 2 |
| Acidimicrobiaceae_Unclassified | 3 | 2 | 2 | 3 | 4 | 7 | 4 | 7 | 2 | 3 | 1 | 3 |
| Acidimicrobiaceae_uncultured | 7 | 21 | 12 | 15 | 38 | 19 | 4 | 15 | 17 | 12 | 18 | 14 |
| Acidimicrobiales_Unclassified | 6 | 21 | 13 | 12 | 13 | 6 | 6 | 16 | 18 | 21 | 22 | 9 |
| Acidimicrobiales_uncultured | 55 | 125 | 106 | 110 | 231 | 153 | 102 | 143 | 189 | 147 | 102 | 79 |
| Acidiphilium | 0 | 0 | 0 | 1 | 2 | 0 | 1 | 0 | 1 | 0 | 0 | 0 |
| Acidisoma | 14 | 5 | 16 | 0 | 1 | 3 | 0 | 0 | 10 | 91 | 4 | 4 |
| Acidobacteria_Unclassified | 0 | 1 | 0 | 0 | 2 | 1 | 0 | 0 | 1 | 0 | 0 | 0 |
| Acidobacteria_norank | 3 | 11 | 4 | 4 | 11 | 9 | 5 | 1 | 5 | 6 | 0 | 3 |
| Acidobacteriaceae (Subgroup 1)_uncultured | 67 | 158 | 135 | 165 | 304 | 242 | 150 | 183 | 262 | 195 | 137 | 69 |
| Acidobacterium | 8 | 18 | 38 | 40 | 7 | 19 | 59 | 43 | 10 | 58 | 12 | 5 |
| Acidothermus | 2 | 10 | 11 | 8 | 12 | 7 | 7 | 4 | 7 | 10 | 3 | 3 |
| Acinetobacter | 4 | 23 | 10 | 1 | 47 | 84 | 47 | 71 | 5 | 7 | 10 | 55 |
| Actinoallomurus | 0 | 1 | 1 | 3 | 1 | 1 | 0 | 2 | 2 | 1 | 0 | 2 |
| Actinobacteria_Unclassified | 1 | 2 | 4 | 3 | 17 | 6 | 3 | 3 | 2 | 4 | 4 | 2 |
| Actinobacteria_norank | 105 | 197 | 196 | 178 | 548 | 318 | 171 | 219 | 297 | 224 | 186 | 152 |
| Actinocorallia | 11 | 10 | 22 | 17 | 39 | 18 | 9 | 9 | 19 | 39 | 12 | 16 |
| Actinomadura | 0 | 1 | 3 | 0 | 0 | 0 | 1 | 0 | 0 | 5 | 2 | 1 |
| Actinomycetospora | 0 | 1 | 2 | 4 | 6 | 7 | 5 | 4 | 6 | 1 | 2 | 5 |
| Actinoplanes | 66 | 41 | 32 | 38 | 151 | 91 | 60 | 64 | 135 | 96 | 75 | 60 |
| Actinospica | 18 | 51 | 16 | 7 | 11 | 3 | 12 | 47 | 4 | 39 | 25 | 8 |
| Adhaeribacter | 6 | 3 | 1 | 6 | 12 | 6 | 5 | 6 | 20 | 2 | 6 | 4 |
| Advenella | 3 | 2 | 1 | 2 | 0 | 1 | 3 | 6 | 3 | 5 | 3 | 1 |
| Aeromicrobium | 256 | 2389 | 261 | 111 | 70 | 67 | 61 | 107 | 1237 | 210 | 143 | 91 |
| Aeromonas | 0 | 5 | 0 | 0 | 13 | 1 | 2 | 20 | 12 | 0 | 1 | 1 |
| Aerophobetes_norank | 1 | 2 | 2 | 1 | 4 | 7 | 6 | 4 | 3 | 6 | 0 | 2 |
| Aestuariimicrobium | 0 | 1 | 1 | 0 | 2 | 0 | 1 | 4 | 0 | 0 | 0 | 0 |
| Afipia | 15 | 14 | 54 | 57 | 29 | 39 | 100 | 113 | 24 | 18 | 40 | 6 |
| Agromyces | 16 | 11 | 7 | 15 | 17 | 16 | 10 | 16 | 31 | 16 | 5 | 11 |
| Alcaligenaceae_Unclassified | 3 | 2 | 2 | 4 | 11 | 43 | 69 | 80 | 57 | 44 | 39 | 12 |
| Alcaligenaceae_uncultured | 0 | 1 | 1601 | 38 | 1 | 3 | 1 | 0 | 2 | 0 | 0 | 0 |
| Alicyclobacillus | 1 | 1 | 2 | 0 | 0 | 0 | 1 | 4 | 0 | 1 | 4 | 4 |
| Allocatelliglobosispora | 0 | 0 | 2 | 1 | 4 | 3 | 2 | 1 | 0 | 1 | 1 | 1 |
| Alphaproteobacteria_Unclassified | 21 | 41 | 29 | 26 | 30 | 43 | 11 | 33 | 56 | 35 | 8 | 8 |
| Alsobacter | 0 | 3 | 1 | 0 | 4 | 4 | 3 | 8 | 2 | 0 | 2 | 0 |
| Altererythrobacter | 12 | 47 | 24 | 24 | 28 | 25 | 17 | 22 | 39 | 31 | 21 | 20 |
| Alterococcus | 6 | 3 | 2 | 2 | 11 | 8 | 5 | 4 | 14 | 7 | 0 | 1 |
| Amaricoccus | 1 | 3 | 5 | 1 | 1 | 0 | 1 | 5 | 2 | 0 | 1 | 2 |
| Aminobacter | 1 | 3 | 11 | 9 | 6 | 2 | 29 | 50 | 0 | 2 | 10 | 7 |
| Amycolatopsis | 15 | 4 | 18 | 18 | 12 | 18 | 7 | 14 | 12 | 23 | 20 | 12 |
| Anaerobacterium | 5 | 4 | 1 | 7 | 15 | 20 | 6 | 7 | 26 | 6 | 7 | 5 |
| Anaerolinea | 4 | 8 | 3 | 12 | 20 | 21 | 18 | 12 | 14 | 9 | 6 | 2 |
| Anaerolineaceae_Unclassified | 4 | 9 | 8 | 6 | 12 | 14 | 5 | 8 | 15 | 11 | 12 | 0 |
| Anaerolineaceae_uncultured | 585 | 1602 | 579 | 742 | 1321 | 1567 | 739 | 816 | 1508 | 1309 | 612 | 467 |
| Anaeromyxobacter | 6 | 10 | 6 | 11 | 19 | 35 | 28 | 10 | 24 | 4 | 2 | 3 |
| Anaerospora | 1 | 0 | 1 | 2 | 9 | 77 | 26 | 4 | 38 | 4 | 0 | 1 |
| Anaerovorax | 0 | 0 | 0 | 0 | 0 | 14 | 6 | 0 | 0 | 1 | 0 | 0 |
| Ancalomicrobium | 1 | 2 | 0 | 0 | 15 | 158 | 85 | 8 | 51 | 21 | 3 | 0 |
| Angustibacter | 0 | 0 | 0 | 4 | 5 | 1 | 14 | 39 | 1 | 0 | 10 | 6 |
| Aquicella | 94 | 126 | 53 | 77 | 218 | 252 | 119 | 101 | 158 | 113 | 78 | 43 |
| Aquincola | 1570 | 386 | 452 | 494 | 1084 | 1573 | 1671 | 1459 | 1106 | 727 | 255 | 699 |
| Arcicella | 0 | 0 | 0 | 0 | 0 | 0 | 0 | 0 | 16 | 0 | 0 | 0 |
| Arcticibacter | 1 | 0 | 0 | 0 | 0 | 13 | 4 | 2 | 20 | 3 | 1 | 0 |
| Ardenticatenales_norank | 1 | 6 | 1 | 1 | 2 | 3 | 1 | 1 | 1 | 2 | 5 | 3 |
| Ardenticatenia_uncultured | 5 | 9 | 4 | 1 | 6 | 6 | 5 | 5 | 7 | 12 | 2 | 1 |
| Arenimonas | 138 | 264 | 117 | 113 | 294 | 438 | 299 | 323 | 292 | 144 | 157 | 94 |
| Armatimonadales_norank | 22 | 22 | 9 | 4 | 56 | 35 | 25 | 27 | 32 | 27 | 9 | 9 |
| Armatimonadetes_norank | 111 | 260 | 125 | 123 | 280 | 348 | 182 | 228 | 312 | 240 | 132 | 80 |
| Armatimonas | 25 | 14 | 16 | 10 | 51 | 49 | 22 | 34 | 22 | 42 | 8 | 4 |
| Arthrobacter | 248 | 449 | 609 | 769 | 465 | 374 | 322 | 524 | 817 | 490 | 481 | 455 |
| Asteroleplasma | 57 | 78 | 81 | 95 | 97 | 17 | 45 | 24 | 18 | 22 | 43 | 22 |
| Asticcacaulis | 268 | 123 | 118 | 244 | 406 | 382 | 336 | 433 | 427 | 209 | 180 | 63 |
| Azoarcus | 9 | 4 | 7 | 21 | 33 | 1301 | 86 | 55 | 34 | 26 | 17 | 4 |
| Azospira | 6 | 1 | 1 | 43 | 576 | 120 | 77 | 7 | 350 | 35 | 2 | 0 |
| Azospirillum | 8 | 11 | 5 | 7 | 65 | 185 | 51 | 40 | 82 | 47 | 26 | 6 |
| Azotobacter | 1 | 0 | 0 | 0 | 0 | 43 | 0 | 1 | 0 | 0 | 0 | 1 |
| Azovibrio | 0 | 0 | 0 | 0 | 22 | 155 | 36 | 2 | 38 | 1 | 0 | 0 |
| B1-7BS_norank | 1 | 2 | 1 | 0 | 6 | 4 | 6 | 1 | 6 | 10 | 1 | 2 |
| B79_norank | 5 | 4 | 0 | 4 | 6 | 8 | 5 | 4 | 7 | 11 | 6 | 2 |
| BCf3-20_norank | 2 | 0 | 3 | 5 | 2 | 3 | 3 | 5 | 5 | 3 | 2 | 3 |
| BD1-7 clade | 5 | 3 | 2 | 0 | 5 | 4 | 3 | 2 | 2 | 4 | 1 | 1 |
| BD2-11 terrestrial group_norank | 4 | 4 | 2 | 7 | 12 | 7 | 5 | 4 | 12 | 7 | 6 | 4 |
| BD7-11_norank | 1 | 7 | 1 | 7 | 18 | 13 | 6 | 3 | 15 | 10 | 1 | 4 |
| BG.g7_norank | 0 | 2 | 0 | 0 | 1 | 3 | 1 | 0 | 4 | 2 | 0 | 0 |
| BIrii41_norank | 51 | 59 | 45 | 33 | 164 | 108 | 46 | 44 | 91 | 64 | 47 | 33 |
| BSV26_norank | 2 | 6 | 3 | 4 | 10 | 7 | 0 | 5 | 15 | 7 | 6 | 8 |
| BVA18_norank | 0 | 0 | 0 | 0 | 4 | 9 | 13 | 1 | 69 | 0 | 0 | 0 |
| Bacillaceae_Unclassified | 14 | 8 | 15 | 25 | 46 | 18 | 22 | 19 | 33 | 10 | 19 | 26 |
| Bacillales_Unclassified | 31 | 74 | 106 | 172 | 70 | 50 | 31 | 36 | 59 | 87 | 47 | 43 |
| Bacillus | 55 | 178 | 282 | 897 | 80 | 65 | 44 | 71 | 163 | 190 | 206 | 205 |
| Bacteria_Unclassified | 165 | 257 | 142 | 166 | 387 | 387 | 344 | 440 | 367 | 377 | 215 | 113 |
| Bacteriovorax | 3 | 1 | 0 | 2 | 4 | 27 | 23 | 26 | 12 | 6 | 2 | 1 |
| Bacteroidales_Unclassified | 0 | 0 | 0 | 0 | 4 | 20 | 3 | 2 | 11 | 4 | 0 | 0 |
| Bacteroidetes VC2.1 Bac22_norank | 6 | 4 | 1 | 0 | 15 | 28 | 9 | 8 | 6 | 11 | 5 | 1 |
| Bacteroidetes_Unclassified | 12 | 16 | 8 | 6 | 57 | 45 | 23 | 8 | 16 | 48 | 4 | 3 |
| Bauldia | 13 | 25 | 25 | 25 | 40 | 29 | 15 | 18 | 43 | 22 | 15 | 10 |
| Bdellovibrio | 4 | 0 | 1 | 2 | 13 | 7 | 8 | 15 | 15 | 4 | 3 | 1 |
| Beijerinckiaceae_Unclassified | 3 | 2 | 4 | 3 | 8 | 6 | 7 | 2 | 13 | 4 | 0 | 1 |
| Beijerinckiaceae_uncultured | 0 | 0 | 0 | 0 | 0 | 41 | 0 | 0 | 8 | 0 | 0 | 0 |
| Betaproteobacteria_Unclassified | 151 | 357 | 154 | 144 | 200 | 226 | 195 | 208 | 176 | 279 | 231 | 196 |
| Blastocatella | 108 | 234 | 81 | 110 | 270 | 422 | 273 | 291 | 275 | 237 | 149 | 72 |
| Blastococcus | 22 | 28 | 38 | 48 | 57 | 39 | 39 | 42 | 69 | 45 | 33 | 35 |
| Blastopirellula | 1 | 3 | 2 | 4 | 4 | 3 | 5 | 2 | 3 | 2 | 1 | 1 |
| Blfdi19_norank | 15 | 16 | 17 | 16 | 61 | 44 | 23 | 14 | 31 | 29 | 12 | 4 |
| Blvii28 wastewater-sludge group | 0 | 0 | 0 | 1 | 1 | 0 | 1 | 0 | 8 | 0 | 0 | 0 |
| Bosea | 17 | 16 | 14 | 8 | 35 | 67 | 48 | 77 | 55 | 36 | 30 | 19 |
| Bradyrhizobiaceae_uncultured | 23 | 33 | 24 | 29 | 49 | 56 | 48 | 31 | 57 | 28 | 40 | 22 |
| Bradyrhizobium | 1606 | 1376 | 1861 | 834 | 635 | 571 | 477 | 605 | 4192 | 535 | 582 | 293 |
| Brevibacillus | 0 | 0 | 0 | 0 | 0 | 1 | 0 | 1 | 2 | 0 | 1 | 1 |
| Brevundimonas | 8 | 13 | 6 | 3 | 5 | 32 | 12 | 7 | 8 | 10 | 22 | 11 |
| Bryobacter | 89 | 198 | 124 | 148 | 293 | 314 | 189 | 174 | 306 | 210 | 121 | 91 |
| Burkholderia | 3834 | 10753 | 4682 | 8568 | 1369 | 1583 | 8451 | 12253 | 1285 | 9691 | 13047 | 12090 |
| Burkholderiaceae_Unclassified | 280 | 1044 | 127 | 66 | 127 | 117 | 1312 | 188 | 18 | 126 | 68 | 53 |
| Burkholderiales_Unclassified | 0 | 1 | 2 | 1 | 3 | 5 | 1 | 0 | 3 | 4 | 1 | 2 |
| Byssovorax | 34 | 48 | 22 | 34 | 62 | 45 | 45 | 23 | 62 | 41 | 22 | 15 |
| C0119_norank | 50 | 111 | 80 | 100 | 150 | 180 | 149 | 177 | 128 | 121 | 93 | 63 |
| C1-B045 | 0 | 0 | 0 | 0 | 0 | 0 | 0 | 2 | 1 | 2 | 1 | 0 |
| C47_norank | 0 | 0 | 0 | 0 | 3 | 1 | 0 | 0 | 1 | 0 | 0 | 0 |
| CHAB-XI-27_norank | 2 | 0 | 0 | 6 | 2 | 0 | 2 | 0 | 3 | 1 | 0 | 1 |
| CL500-29 marine group | 24 | 89 | 37 | 60 | 122 | 87 | 58 | 69 | 109 | 67 | 69 | 43 |
| CPla-3 termite group_norank | 22 | 18 | 13 | 19 | 73 | 78 | 35 | 20 | 50 | 23 | 11 | 4 |
| Caldilineaceae_Unclassified | 0 | 1 | 1 | 0 | 0 | 0 | 0 | 1 | 1 | 1 | 1 | 0 |
| Caldilineaceae_norank | 2 | 4 | 1 | 2 | 11 | 7 | 7 | 5 | 12 | 5 | 5 | 1 |
| Caldilineaceae_uncultured | 15 | 41 | 20 | 24 | 47 | 37 | 22 | 16 | 47 | 27 | 22 | 8 |
| Candidate division OP3_norank | 5 | 11 | 4 | 8 | 18 | 8 | 3 | 0 | 21 | 16 | 4 | 4 |
| Candidatus Accumulibacter | 0 | 0 | 0 | 1 | 0 | 7 | 23 | 0 | 9 | 4 | 0 | 1 |
| Candidatus Alysiosphaera | 1 | 5 | 2 | 1 | 7 | 3 | 9 | 4 | 7 | 4 | 1 | 3 |
| Candidatus Amoebophilus | 1 | 0 | 0 | 1 | 0 | 0 | 0 | 0 | 1 | 4 | 0 | 0 |
| Candidatus Aquiluna | 0 | 7 | 3 | 2 | 3 | 2 | 4 | 9 | 2 | 1 | 1 | 0 |
| Candidatus Captivus | 6 | 0 | 7 | 8 | 5 | 0 | 4 | 1 | 10 | 4 | 1 | 0 |
| Candidatus Chloroploca | 0 | 0 | 2 | 0 | 1 | 1 | 0 | 0 | 0 | 1 | 0 | 0 |
| Candidatus Entotheonella | 2 | 8 | 4 | 10 | 17 | 3 | 5 | 12 | 10 | 5 | 5 | 3 |
| Candidatus Glomeribacter | 0 | 4 | 5 | 1 | 5 | 2 | 1 | 2 | 1 | 0 | 1 | 1 |
| Candidatus Koribacter | 3 | 12 | 11 | 8 | 37 | 39 | 27 | 17 | 30 | 21 | 10 | 7 |
| Candidatus Microthrix | 0 | 1 | 1 | 0 | 1 | 1 | 0 | 0 | 1 | 0 | 0 | 0 |
| Candidatus Odyssella | 3 | 4 | 3 | 3 | 7 | 2 | 9 | 4 | 15 | 4 | 5 | 2 |
| Candidatus Paenicardinium | 1 | 0 | 5 | 3 | 5 | 3 | 4 | 3 | 6 | 4 | 22 | 0 |
| Candidatus Pelagibacter | 1 | 2 | 1 | 4 | 4 | 0 | 0 | 0 | 1 | 0 | 0 | 0 |
| Candidatus Planktophila | 3 | 12 | 2 | 7 | 3 | 1 | 1 | 18 | 0 | 5 | 4 | 4 |
| Candidatus Rhabdochlamydia | 0 | 0 | 0 | 0 | 1 | 0 | 0 | 0 | 1 | 0 | 0 | 0 |
| Candidatus Riegeria | 0 | 1 | 0 | 0 | 15 | 6 | 0 | 0 | 0 | 0 | 1 | 0 |
| Candidatus Solibacter | 43 | 100 | 70 | 88 | 170 | 130 | 78 | 51 | 136 | 94 | 68 | 37 |
| Castellaniella | 3 | 3 | 122 | 259 | 15 | 9 | 135 | 191 | 5 | 9 | 190 | 213 |
| Catenulispora | 0 | 0 | 0 | 0 | 0 | 1 | 1 | 0 | 0 | 0 | 2 | 0 |
| Caulobacter | 149 | 72 | 59 | 40 | 203 | 165 | 145 | 327 | 300 | 187 | 165 | 63 |
| Caulobacteraceae_Unclassified | 18 | 6 | 8 | 20 | 80 | 49 | 28 | 36 | 12 | 21 | 8 | 6 |
| Caulobacteraceae_uncultured | 96 | 36 | 45 | 43 | 193 | 86 | 95 | 90 | 145 | 55 | 43 | 41 |
| Cellulosimicrobium | 0 | 1 | 2 | 2 | 3 | 10 | 10 | 5 | 9 | 0 | 3 | 1 |
| Cellvibrio | 577 | 122 | 88 | 89 | 324 | 171 | 75 | 90 | 368 | 95 | 497 | 213 |
| Chelatococcus | 4 | 1 | 3 | 10 | 6 | 4 | 5 | 6 | 4 | 0 | 2 | 3 |
| Chitinimonas | 1 | 0 | 1 | 0 | 0 | 0 | 0 | 0 | 7 | 0 | 0 | 0 |
| Chitinophaga | 305 | 262 | 138 | 199 | 301 | 575 | 304 | 482 | 543 | 413 | 302 | 149 |
| Chitinophagaceae_Unclassified | 192 | 237 | 99 | 109 | 386 | 385 | 197 | 224 | 428 | 295 | 208 | 59 |
| Chitinophagaceae_uncultured | 214 | 268 | 334 | 460 | 336 | 664 | 716 | 1103 | 433 | 479 | 410 | 231 |
| Chlamydiales_Unclassified | 2 | 1 | 0 | 0 | 0 | 0 | 0 | 0 | 3 | 2 | 2 | 0 |
| Chloroflexaceae_Unclassified | 1 | 1 | 2 | 0 | 0 | 2 | 1 | 4 | 7 | 2 | 0 | 0 |
| Chloroflexi_Unclassified | 2 | 4 | 4 | 8 | 8 | 3 | 5 | 3 | 6 | 7 | 1 | 0 |
| Chloroflexi_norank | 7 | 11 | 11 | 12 | 24 | 19 | 10 | 16 | 21 | 26 | 5 | 6 |
| Chloroflexi_uncultured | 23 | 80 | 30 | 50 | 119 | 86 | 52 | 46 | 83 | 89 | 52 | 28 |
| Chloronema | 0 | 4 | 2 | 3 | 4 | 1 | 0 | 1 | 5 | 1 | 3 | 1 |
| Christensenellaceae R-7 group | 0 | 0 | 0 | 0 | 2 | 0 | 0 | 0 | 5 | 0 | 0 | 0 |
| Chryseobacterium | 41 | 47 | 8 | 1 | 31 | 33 | 46 | 199 | 457 | 80 | 98 | 15 |
| Chryseolinea | 12 | 8 | 2 | 1 | 8 | 10 | 6 | 1 | 11 | 6 | 9 | 1 |
| Chthoniobacter | 2 | 13 | 2 | 2 | 16 | 18 | 9 | 23 | 24 | 5 | 3 | 5 |
| Chthonomonadales_norank | 19 | 104 | 40 | 48 | 96 | 117 | 49 | 40 | 80 | 107 | 38 | 22 |
| Chthonomonas | 15 | 49 | 16 | 30 | 54 | 56 | 22 | 39 | 45 | 55 | 23 | 14 |
| Cloacibacterium | 2 | 3 | 0 | 2 | 9 | 186 | 68 | 4 | 49 | 6 | 0 | 1 |
| Clostridiales_Unclassified | 0 | 0 | 1 | 0 | 0 | 0 | 0 | 1 | 0 | 0 | 0 | 3 |
| Clostridium sensu stricto 1 | 2 | 1 | 4 | 5 | 22 | 17 | 20 | 8 | 12 | 1 | 6 | 2 |
| Clostridium sensu stricto 10 | 0 | 0 | 0 | 0 | 2 | 7 | 5 | 1 | 3 | 0 | 1 | 0 |
| Clostridium sensu stricto 12 | 0 | 2 | 2 | 1 | 7 | 12 | 6 | 5 | 6 | 0 | 1 | 2 |
| Clostridium sensu stricto 13 | 6 | 1 | 6 | 3 | 20 | 12 | 8 | 10 | 9 | 9 | 12 | 2 |
| Clostridium sensu stricto 3 | 0 | 0 | 0 | 0 | 1 | 1 | 4 | 0 | 1 | 1 | 1 | 0 |
| Clostridium sensu stricto 8 | 1 | 2 | 1 | 0 | 3 | 17 | 11 | 1 | 2 | 4 | 0 | 0 |
| Cohnella | 1 | 1 | 5 | 52 | 5 | 3 | 34 | 13 | 5 | 7 | 49 | 13 |
| Comamonadaceae_Unclassified | 352 | 201 | 204 | 288 | 307 | 335 | 285 | 220 | 398 | 298 | 131 | 165 |
| Comamonadaceae_uncultured | 6 | 0 | 0 | 8 | 95 | 46 | 324 | 11 | 29 | 2 | 16 | 3 |
| Conexibacter | 2 | 7 | 2 | 3 | 7 | 2 | 4 | 6 | 3 | 4 | 0 | 7 |
| Coriobacteriaceae_uncultured | 1 | 0 | 0 | 0 | 0 | 11 | 12 | 0 | 6 | 1 | 0 | 0 |
| Coxiella | 4 | 7 | 4 | 4 | 8 | 5 | 3 | 0 | 5 | 1 | 1 | 2 |
| Coxiellaceae_Unclassified | 1 | 0 | 1 | 0 | 3 | 1 | 1 | 1 | 0 | 6 | 0 | 0 |
| Coxiellaceae_uncultured | 8 | 6 | 5 | 6 | 9 | 10 | 0 | 2 | 13 | 11 | 4 | 1 |
| Crocinitomix | 8 | 0 | 4 | 8 | 35 | 4 | 11 | 1 | 45 | 4 | 16 | 1 |
| Cryptosporangium | 3 | 1 | 0 | 0 | 3 | 1 | 0 | 0 | 2 | 2 | 2 | 1 |
| Cupriavidus | 7 | 2 | 10 | 9 | 17 | 17 | 54 | 31 | 34 | 15 | 11 | 9 |
| Cyanobacteria_Unclassified | 12 | 32 | 68 | 9 | 59 | 51 | 39 | 19 | 39 | 85 | 29 | 30 |
| Cyanobacteria_norank | 39787 | 24193 | 36029 | 41296 | 26864 | 6108 | 12794 | 8906 | 14762 | 12859 | 16781 | 29079 |
| Cyanothece | 0 | 0 | 0 | 0 | 0 | 0 | 0 | 2 | 0 | 0 | 0 | 0 |
| Cyclobacteriaceae_Unclassified | 4 | 14 | 1 | 9 | 7 | 3 | 1 | 1 | 5 | 0 | 1 | 4 |
| Cyclobacteriaceae_uncultured | 15 | 11 | 6 | 4 | 8 | 7 | 5 | 7 | 14 | 7 | 8 | 2 |
| Cystobacter | 75 | 127 | 83 | 109 | 323 | 184 | 135 | 105 | 179 | 120 | 81 | 120 |
| Cytophaga | 65 | 10 | 10 | 10 | 96 | 106 | 35 | 4 | 79 | 28 | 6 | 7 |
| Cytophagaceae_Unclassified | 22 | 71 | 36 | 56 | 30 | 28 | 14 | 20 | 44 | 17 | 19 | 7 |
| Cytophagaceae_uncultured | 297 | 269 | 146 | 121 | 275 | 248 | 134 | 102 | 466 | 138 | 121 | 63 |
| Cytophagales_Unclassified | 85 | 5 | 6 | 4 | 49 | 21 | 2 | 1 | 56 | 19 | 11 | 14 |
| DA101 soil group_norank | 7 | 40 | 15 | 9 | 53 | 32 | 25 | 35 | 62 | 24 | 17 | 8 |
| DA111_norank | 8 | 21 | 16 | 18 | 53 | 31 | 25 | 32 | 44 | 25 | 20 | 17 |
| DB1-14_norank | 32 | 37 | 29 | 52 | 66 | 247 | 37 | 36 | 124 | 63 | 26 | 21 |
| DS-100_norank | 32 | 31 | 18 | 13 | 54 | 92 | 46 | 29 | 54 | 40 | 18 | 24 |
| Dactylosporangium | 22 | 18 | 17 | 25 | 53 | 25 | 34 | 22 | 42 | 27 | 22 | 17 |
| Dechloromonas | 24 | 1 | 5 | 195 | 680 | 2581 | 1091 | 177 | 1535 | 170 | 46 | 10 |
| Defluviicoccus | 4 | 13 | 9 | 10 | 13 | 10 | 10 | 7 | 13 | 9 | 5 | 2 |
| Dehalococcoidales_uncultured | 0 | 0 | 0 | 0 | 0 | 0 | 0 | 1 | 0 | 0 | 0 | 1 |
| Deinococcus | 0 | 2 | 0 | 2 | 3 | 10 | 5 | 6 | 4 | 6 | 2 | 1 |
| Delftia | 15 | 17 | 19 | 31 | 47 | 92 | 51 | 69 | 135 | 32 | 34 | 30 |
| Deltaproteobacteria_Unclassified | 5 | 6 | 3 | 1 | 13 | 11 | 3 | 3 | 4 | 7 | 0 | 2 |
| Demequinaceae_Unclassified | 0 | 0 | 0 | 0 | 1 | 2 | 1 | 0 | 1 | 1 | 0 | 0 |
| Derxia | 0 | 2 | 8 | 9 | 4 | 1 | 2 | 0 | 1 | 0 | 0 | 0 |
| Desulfobulbus | 0 | 0 | 0 | 0 | 1 | 8 | 6 | 0 | 5 | 0 | 0 | 0 |
| Desulfocapsa | 0 | 0 | 0 | 2 | 0 | 8 | 10 | 0 | 12 | 0 | 0 | 0 |
| Desulfosporosinus | 1 | 4 | 0 | 8 | 18 | 17 | 5 | 2 | 8 | 2 | 3 | 1 |
| Desulfovibrio | 0 | 0 | 0 | 7 | 39 | 86 | 32 | 4 | 107 | 9 | 0 | 0 |
| Desulfurellaceae_uncultured | 3 | 15 | 17 | 24 | 30 | 24 | 16 | 14 | 31 | 12 | 13 | 8 |
| Desulfuromonadales_Unclassified | 0 | 0 | 0 | 1 | 0 | 2 | 17 | 3 | 2 | 0 | 0 | 0 |
| Devosia | 280 | 358 | 356 | 410 | 368 | 472 | 432 | 545 | 456 | 529 | 357 | 193 |
| Dokdonella | 15 | 8 | 3 | 7 | 38 | 28 | 23 | 19 | 7 | 8 | 18 | 7 |
| Dongia | 391 | 223 | 132 | 191 | 444 | 321 | 165 | 243 | 365 | 237 | 136 | 89 |
| Draconibacteriaceae_uncultured | 0 | 0 | 0 | 0 | 8 | 2 | 0 | 1 | 0 | 0 | 0 | 0 |
| Dyadobacter | 71 | 110 | 104 | 125 | 49 | 130 | 74 | 201 | 277 | 121 | 139 | 83 |
| Dyella | 750 | 1966 | 610 | 376 | 611 | 566 | 879 | 1081 | 446 | 4342 | 2149 | 355 |
| EV818SWSAP88_norank | 0 | 0 | 0 | 0 | 0 | 1 | 0 | 0 | 1 | 0 | 0 | 0 |
| Edaphobacter | 8 | 63 | 10 | 7 | 30 | 30 | 76 | 121 | 13 | 126 | 32 | 3 |
| Elev-16S-1158_norank | 3 | 17 | 8 | 8 | 14 | 21 | 6 | 9 | 15 | 7 | 2 | 6 |
| Elev-16S-1166_norank | 5 | 11 | 6 | 11 | 14 | 35 | 7 | 8 | 18 | 18 | 11 | 4 |
| Elev-16S-1332_norank | 3 | 11 | 1 | 5 | 11 | 12 | 4 | 11 | 6 | 4 | 6 | 4 |
| Emticicia | 0 | 1 | 1 | 1 | 3 | 11 | 5 | 5 | 13 | 2 | 2 | 0 |
| Enterobacteriaceae_Unclassified | 10 | 7 | 1 | 0 | 61 | 141 | 343 | 586 | 132 | 110 | 47 | 146 |
| Erysipelothrix | 0 | 0 | 0 | 0 | 0 | 0 | 0 | 0 | 4 | 0 | 0 | 0 |
| Erythrobacteraceae_Unclassified | 8 | 34 | 20 | 19 | 20 | 12 | 9 | 18 | 5 | 18 | 7 | 12 |
| Erythrobacteraceae_uncultured | 7 | 18 | 23 | 18 | 33 | 21 | 44 | 72 | 26 | 14 | 25 | 12 |
| Exiguobacterium | 1 | 0 | 2 | 0 | 1 | 3 | 2 | 2 | 2 | 2 | 1 | 2 |
| FCPU744_norank | 0 | 3 | 2 | 2 | 0 | 0 | 0 | 2 | 5 | 0 | 0 | 3 |
| FFCH13075_norank | 2 | 5 | 5 | 3 | 6 | 5 | 1 | 4 | 3 | 4 | 7 | 7 |
| FFCH7168_norank | 20 | 35 | 21 | 20 | 58 | 56 | 37 | 31 | 57 | 40 | 19 | 13 |
| Family XIII_Unclassified | 1 | 0 | 0 | 2 | 0 | 0 | 3 | 1 | 10 | 0 | 0 | 0 |
| Family XVIII_Unclassified | 1 | 0 | 0 | 0 | 0 | 0 | 1 | 0 | 0 | 1 | 0 | 0 |
| Ferrovibrio | 12 | 10 | 6 | 7 | 19 | 9 | 3 | 1 | 7 | 5 | 3 | 3 |
| Ferruginibacter | 32 | 59 | 19 | 31 | 39 | 111 | 52 | 59 | 99 | 47 | 46 | 12 |
| Fibrisoma | 0 | 1 | 0 | 1 | 0 | 7 | 0 | 0 | 0 | 0 | 0 | 1 |
| Fibrobacteraceae_uncultured | 0 | 0 | 0 | 0 | 3 | 5 | 3 | 0 | 2 | 0 | 0 | 0 |
| Filimonas | 2 | 3 | 0 | 0 | 1 | 3 | 3 | 9 | 1 | 1 | 1 | 2 |
| Flavihumibacter | 5 | 1 | 2 | 6 | 5 | 11 | 2 | 5 | 0 | 7 | 2 | 1 |
| Flavisolibacter | 111 | 200 | 136 | 227 | 213 | 537 | 273 | 346 | 275 | 281 | 159 | 102 |
| Flavitalea | 28 | 54 | 32 | 52 | 34 | 55 | 25 | 52 | 46 | 28 | 33 | 24 |
| Flavobacterium | 198 | 163 | 193 | 154 | 147 | 146 | 251 | 343 | 1754 | 198 | 433 | 230 |
| Flectobacillus | 2 | 2 | 0 | 0 | 9 | 0 | 1 | 1 | 13 | 15 | 7 | 4 |
| Flexibacter | 0 | 2 | 0 | 0 | 2 | 0 | 1 | 0 | 0 | 2 | 0 | 0 |
| Flexivirga | 0 | 0 | 1 | 0 | 0 | 1 | 1 | 8 | 1 | 0 | 2 | 1 |
| Fluviicola | 443 | 50 | 50 | 45 | 414 | 235 | 141 | 257 | 547 | 98 | 222 | 60 |
| Fonticella | 0 | 0 | 2 | 1 | 2 | 1 | 2 | 2 | 3 | 2 | 3 | 1 |
| Fontimonas | 3 | 9 | 3 | 4 | 12 | 7 | 4 | 9 | 13 | 8 | 6 | 5 |
| Formivibrio | 0 | 1 | 0 | 0 | 3 | 7 | 4 | 0 | 5 | 0 | 0 | 0 |
| Frankiales_Unclassified | 4 | 2 | 2 | 1 | 10 | 3 | 9 | 11 | 2 | 2 | 1 | 5 |
| Frankiales_uncultured | 2 | 23 | 9 | 8 | 15 | 30 | 37 | 89 | 25 | 14 | 24 | 30 |
| Friedmanniella | 15 | 20 | 18 | 26 | 48 | 31 | 25 | 27 | 62 | 28 | 34 | 24 |
| GR-WP33-30_norank | 73 | 192 | 89 | 153 | 365 | 272 | 133 | 147 | 257 | 186 | 124 | 73 |
| GR-WP33-58_norank | 2 | 0 | 1 | 2 | 11 | 3 | 1 | 2 | 3 | 3 | 3 | 1 |
| Gaiella | 155 | 257 | 258 | 270 | 735 | 461 | 286 | 348 | 391 | 357 | 242 | 193 |
| Gaiellales_Unclassified | 7 | 10 | 5 | 8 | 22 | 15 | 18 | 8 | 7 | 15 | 4 | 5 |
| Gaiellales_uncultured | 175 | 251 | 255 | 273 | 618 | 370 | 299 | 384 | 358 | 340 | 226 | 210 |
| Gallionellaceae_Unclassified | 0 | 0 | 0 | 0 | 3 | 0 | 0 | 1 | 54 | 1 | 0 | 0 |
| Gammaproteobacteria_Unclassified | 13 | 19 | 9 | 23 | 27 | 10 | 10 | 9 | 47 | 18 | 8 | 5 |
| Geminicoccus | 0 | 1 | 1 | 1 | 3 | 3 | 1 | 1 | 7 | 1 | 3 | 2 |
| Gemmata | 44 | 59 | 42 | 49 | 107 | 101 | 70 | 76 | 89 | 71 | 40 | 21 |
| Gemmatimonadaceae_Unclassified | 11 | 23 | 12 | 22 | 24 | 26 | 13 | 18 | 34 | 26 | 19 | 12 |
| Gemmatimonadaceae_uncultured | 233 | 499 | 318 | 435 | 882 | 702 | 378 | 475 | 740 | 517 | 330 | 198 |
| Gemmatimonas | 97 | 152 | 140 | 175 | 322 | 276 | 160 | 196 | 279 | 209 | 132 | 109 |
| Geobacter | 7 | 5 | 3 | 31 | 68 | 289 | 175 | 20 | 390 | 43 | 11 | 4 |
| Geodermatophilus | 3 | 3 | 6 | 10 | 10 | 6 | 6 | 7 | 7 | 3 | 4 | 3 |
| Geothrix | 2 | 2 | 0 | 13 | 16 | 18 | 61 | 2 | 116 | 6 | 2 | 0 |
| Gitt-GS-136_norank | 32 | 40 | 26 | 29 | 136 | 81 | 36 | 59 | 59 | 78 | 33 | 26 |
| Glycomyces | 0 | 3 | 1 | 4 | 2 | 4 | 0 | 5 | 2 | 12 | 1 | 6 |
| Gordonia | 0 | 0 | 2 | 0 | 5 | 19 | 23 | 20 | 2 | 1 | 2 | 2 |
| Gracilibacter | 0 | 0 | 0 | 0 | 6 | 4 | 2 | 1 | 9 | 0 | 0 | 0 |
| Gracilibacteria_norank | 3 | 3 | 2 | 2 | 10 | 19 | 48 | 3 | 157 | 3 | 0 | 0 |
| Granulicella | 15 | 41 | 75 | 18 | 38 | 33 | 142 | 131 | 41 | 151 | 153 | 13 |
| Gryllotalpicola | 1 | 0 | 3 | 0 | 6 | 3 | 14 | 1 | 4 | 25 | 2 | 1 |
| HSB OF53-F07_norank | 1 | 8 | 1 | 5 | 2 | 1 | 2 | 5 | 5 | 3 | 2 | 3 |
| Hahella | 0 | 6 | 0 | 0 | 1 | 0 | 0 | 1 | 0 | 0 | 0 | 3 |
| Haliangium | 171 | 309 | 147 | 147 | 478 | 411 | 256 | 233 | 352 | 285 | 156 | 98 |
| Haliscomenobacter | 1 | 3 | 1 | 8 | 4 | 1 | 3 | 0 | 49 | 3 | 0 | 1 |
| Haloferula | 0 | 2 | 1 | 0 | 1 | 1 | 3 | 0 | 6 | 0 | 0 | 0 |
| Halomonas | 7 | 45 | 27 | 36 | 75 | 48 | 24 | 100 | 40 | 81 | 31 | 11 |
| Herbaspirillum | 198 | 60 | 68 | 136 | 97 | 126 | 151 | 219 | 189 | 139 | 97 | 109 |
| Herbidospora | 0 | 1 | 0 | 0 | 1 | 2 | 0 | 0 | 0 | 4 | 0 | 0 |
| Herpetosiphon | 9 | 30 | 12 | 29 | 7 | 12 | 12 | 6 | 21 | 23 | 9 | 5 |
| Hirschia | 0 | 5 | 0 | 0 | 5 | 2 | 3 | 3 | 6 | 2 | 1 | 0 |
| Holosporaceae_uncultured | 12 | 7 | 12 | 6 | 22 | 9 | 17 | 7 | 19 | 4 | 4 | 3 |
| Hydrogenedentes_norank | 3 | 11 | 4 | 3 | 19 | 17 | 16 | 23 | 28 | 14 | 5 | 7 |
| Hymenobacter | 1 | 3 | 1 | 0 | 3 | 0 | 2 | 3 | 4 | 1 | 1 | 0 |
| Hyphomicrobiaceae_Unclassified | 1 | 7 | 6 | 4 | 12 | 2 | 18 | 12 | 10 | 4 | 3 | 2 |
| Hyphomicrobiaceae_uncultured | 0 | 0 | 0 | 0 | 0 | 2 | 15 | 2 | 11 | 1 | 0 | 0 |
| Hyphomicrobium | 104 | 100 | 165 | 163 | 121 | 136 | 202 | 364 | 103 | 91 | 151 | 69 |
| I-10_norank | 19 | 16 | 15 | 28 | 48 | 29 | 15 | 17 | 38 | 32 | 12 | 12 |
| Iamia | 6 | 10 | 12 | 11 | 19 | 11 | 9 | 19 | 6 | 12 | 8 | 11 |
| Ideonella | 204 | 131 | 75 | 88 | 298 | 887 | 631 | 223 | 478 | 235 | 84 | 70 |
| Idiomarina | 0 | 0 | 2 | 2 | 2 | 1 | 0 | 27 | 0 | 0 | 0 | 0 |
| Inhella | 1 | 0 | 0 | 0 | 2 | 1 | 0 | 0 | 2 | 0 | 0 | 2 |
| Inquilinus | 6 | 4 | 9 | 7 | 10 | 8 | 15 | 12 | 9 | 15 | 11 | 10 |
| Intrasporangiaceae_Unclassified | 22 | 49 | 41 | 45 | 80 | 78 | 68 | 129 | 69 | 73 | 68 | 30 |
| Isosphaera | 3 | 4 | 2 | 3 | 11 | 4 | 9 | 6 | 6 | 2 | 1 | 1 |
| JG30-KF-CM45_norank | 32 | 51 | 49 | 61 | 92 | 84 | 66 | 101 | 93 | 60 | 33 | 45 |
| JG30-KF-CM66_norank | 0 | 1 | 2 | 4 | 13 | 11 | 6 | 1 | 7 | 9 | 6 | 4 |
| JG30a-KF-32_norank | 1 | 1 | 1 | 2 | 0 | 1 | 1 | 3 | 1 | 1 | 1 | 0 |
| JG34-KF-161_norank | 23 | 58 | 17 | 16 | 74 | 37 | 35 | 92 | 78 | 70 | 25 | 14 |
| JG34-KF-361_norank | 4 | 20 | 9 | 7 | 14 | 16 | 7 | 7 | 13 | 16 | 3 | 5 |
| JG37-AG-20_norank | 0 | 0 | 1 | 1 | 3 | 1 | 0 | 0 | 3 | 0 | 1 | 0 |
| JL-ETNP-Z39_norank | 0 | 1 | 1 | 1 | 2 | 6 | 2 | 0 | 1 | 2 | 3 | 0 |
| Jatrophihabitans | 4 | 16 | 14 | 5 | 21 | 23 | 45 | 74 | 11 | 25 | 15 | 9 |
| KCM-B-15_norank | 2 | 8 | 2 | 1 | 7 | 12 | 4 | 4 | 6 | 7 | 0 | 1 |
| KCM-B-60_norank | 7 | 10 | 7 | 8 | 20 | 15 | 8 | 7 | 18 | 3 | 3 | 5 |
| KD3-10_norank | 1 | 2 | 1 | 0 | 8 | 5 | 7 | 2 | 2 | 6 | 0 | 1 |
| KD3-93_norank | 1 | 1 | 3 | 2 | 3 | 4 | 1 | 5 | 7 | 1 | 0 | 1 |
| KD4-96_norank | 143 | 248 | 237 | 248 | 616 | 392 | 277 | 308 | 374 | 404 | 211 | 183 |
| KF-JG30-B3_norank | 10 | 28 | 18 | 12 | 25 | 32 | 15 | 15 | 24 | 15 | 18 | 9 |
| Kaistia | 0 | 4 | 0 | 1 | 6 | 14 | 15 | 11 | 5 | 7 | 7 | 1 |
| Kineosporia | 14 | 17 | 25 | 17 | 40 | 31 | 26 | 20 | 39 | 36 | 16 | 19 |
| Kocuria | 9 | 25 | 25 | 26 | 21 | 24 | 28 | 61 | 38 | 17 | 31 | 36 |
| Kribbella | 16 | 26 | 19 | 20 | 30 | 22 | 16 | 27 | 25 | 28 | 19 | 23 |
| Ktedonobacteraceae_Unclassified | 1 | 1 | 0 | 1 | 2 | 4 | 0 | 0 | 1 | 4 | 2 | 0 |
| Ktedonobacteraceae_uncultured | 3 | 4 | 0 | 1 | 1 | 0 | 2 | 4 | 1 | 4 | 4 | 3 |
| Labrys | 31 | 23 | 18 | 25 | 82 | 38 | 47 | 90 | 85 | 32 | 29 | 18 |
| Lachnoclostridium 10 | 0 | 0 | 0 | 0 | 0 | 1 | 3 | 0 | 1 | 0 | 0 | 0 |
| Lachnospiraceae_Unclassified | 0 | 0 | 0 | 0 | 6 | 3 | 3 | 0 | 2 | 0 | 0 | 0 |
| Lacibacter | 41 | 58 | 57 | 51 | 42 | 166 | 50 | 61 | 88 | 58 | 25 | 36 |
| Latescibacteria_norank | 46 | 109 | 72 | 79 | 165 | 123 | 80 | 59 | 148 | 103 | 53 | 51 |
| Lechevalieria | 31 | 44 | 32 | 42 | 60 | 41 | 20 | 76 | 43 | 64 | 36 | 43 |
| Legionella | 37 | 45 | 39 | 50 | 73 | 71 | 88 | 35 | 72 | 39 | 38 | 12 |
| Legionellaceae_Unclassified | 0 | 1 | 2 | 1 | 3 | 0 | 0 | 1 | 0 | 0 | 0 | 0 |
| Legionellales_Unclassified | 2 | 0 | 2 | 3 | 1 | 1 | 0 | 1 | 1 | 0 | 0 | 0 |
| Leifsonia | 38 | 198 | 73 | 63 | 97 | 43 | 198 | 781 | 30 | 323 | 301 | 115 |
| Leptolinea | 0 | 0 | 1 | 3 | 2 | 0 | 3 | 2 | 1 | 1 | 1 | 0 |
| Leptolyngbya | 4 | 33 | 26 | 46 | 79 | 46 | 30 | 18 | 325 | 30 | 30 | 30 |
| Leptospira | 16 | 4 | 4 | 5 | 66 | 8 | 7 | 2 | 25 | 8 | 3 | 2 |
| Leptotrichiaceae_uncultured | 0 | 0 | 0 | 0 | 0 | 0 | 0 | 0 | 31 | 0 | 0 | 0 |
| Limnobacter | 29 | 9 | 5 | 4 | 26 | 1008 | 247 | 31 | 5 | 40 | 3 | 4 |
| Limnothrix | 1 | 0 | 0 | 1 | 0 | 1 | 0 | 0 | 2 | 3 | 1 | 0 |
| Lineage IIa_norank | 3 | 19 | 1 | 7 | 10 | 9 | 3 | 6 | 10 | 13 | 2 | 2 |
| Lineage IIb_norank | 9 | 29 | 5 | 7 | 30 | 19 | 14 | 4 | 22 | 14 | 4 | 8 |
| Lineage IV_norank | 2 | 4 | 3 | 2 | 19 | 0 | 1 | 3 | 9 | 4 | 6 | 0 |
| Litorilinea | 3 | 3 | 4 | 2 | 5 | 2 | 1 | 1 | 3 | 1 | 1 | 3 |
| Luedemannella | 0 | 5 | 0 | 2 | 4 | 6 | 1 | 2 | 2 | 2 | 4 | 2 |
| Luteibacter | 27 | 39 | 11 | 9 | 28 | 65 | 61 | 42 | 30 | 152 | 38 | 21 |
| Luteolibacter | 1 | 7 | 3 | 0 | 0 | 4 | 4 | 8 | 5 | 3 | 5 | 2 |
| Lutispora | 0 | 2 | 1 | 2 | 4 | 7 | 6 | 2 | 6 | 0 | 0 | 1 |
| Lysinimonas | 5 | 15 | 11 | 11 | 44 | 45 | 37 | 47 | 28 | 11 | 25 | 12 |
| Lysobacter | 178 | 264 | 130 | 129 | 346 | 410 | 535 | 504 | 383 | 268 | 248 | 228 |
| M20-Pitesti_norank | 3 | 5 | 0 | 9 | 15 | 148 | 119 | 19 | 315 | 35 | 9 | 0 |
| ML80_norank | 0 | 1 | 0 | 2 | 0 | 3 | 2 | 0 | 0 | 0 | 1 | 0 |
| MNC12_norank | 12 | 12 | 9 | 10 | 26 | 25 | 10 | 17 | 21 | 16 | 6 | 3 |
| MND8_norank | 0 | 0 | 1 | 1 | 5 | 3 | 1 | 2 | 5 | 1 | 1 | 0 |
| MNG7_norank | 53 | 56 | 59 | 45 | 117 | 92 | 61 | 62 | 140 | 49 | 33 | 31 |
| MVP-88_norank | 11 | 9 | 5 | 6 | 41 | 17 | 8 | 6 | 13 | 6 | 2 | 3 |
| MWH-CFBk5_norank | 0 | 0 | 0 | 0 | 1 | 1 | 0 | 0 | 0 | 1 | 0 | 0 |
| Magnetospirillum | 0 | 0 | 0 | 4 | 9 | 11 | 3 | 3 | 23 | 0 | 3 | 1 |
| Mangroviflexus | 0 | 0 | 0 | 0 | 0 | 0 | 0 | 0 | 4 | 0 | 0 | 0 |
| Marinicella | 1 | 0 | 0 | 1 | 2 | 1 | 1 | 0 | 2 | 1 | 1 | 0 |
| Marmoricola | 67 | 114 | 100 | 134 | 202 | 213 | 174 | 330 | 187 | 144 | 172 | 138 |
| Martelella | 0 | 4 | 1 | 0 | 0 | 1 | 1 | 3 | 0 | 0 | 1 | 2 |
| Massilia | 631 | 482 | 426 | 617 | 411 | 522 | 601 | 1314 | 714 | 890 | 1140 | 741 |
| Meganema | 0 | 1 | 0 | 0 | 0 | 0 | 0 | 0 | 2 | 0 | 0 | 0 |
| Meiothermus | 0 | 0 | 0 | 0 | 1 | 0 | 0 | 1 | 0 | 0 | 0 | 0 |
| Mesorhizobium | 190 | 363 | 184 | 252 | 370 | 475 | 356 | 565 | 424 | 288 | 292 | 146 |
| Methylobacillus | 240 | 441 | 445 | 607 | 511 | 627 | 327 | 392 | 455 | 304 | 230 | 175 |
| Methylobacter | 1 | 4 | 1 | 1 | 8 | 3 | 0 | 1 | 2 | 1 | 2 | 0 |
| Methylobacteriaceae_Unclassified | 0 | 0 | 0 | 0 | 2 | 0 | 0 | 0 | 0 | 0 | 0 | 0 |
| Methylobacteriaceae_uncultured | 4 | 4 | 10 | 16 | 11 | 15 | 7 | 13 | 1 | 0 | 1 | 2 |
| Methylobacterium | 14 | 9 | 9 | 13 | 30 | 26 | 15 | 25 | 30 | 35 | 33 | 19 |
| Methylophilaceae_uncultured | 815 | 1074 | 746 | 1244 | 620 | 1514 | 703 | 991 | 1022 | 1116 | 546 | 437 |
| Methylophilus | 6 | 12 | 9 | 8 | 5 | 6 | 4 | 2 | 13 | 1 | 3 | 2 |
| Methylotenera | 276 | 387 | 300 | 361 | 237 | 180 | 248 | 247 | 759 | 228 | 270 | 116 |
| Methyloversatilis | 23 | 9 | 2 | 6 | 27 | 18 | 5 | 2 | 76 | 17 | 2 | 3 |
| Microbacteriaceae_Unclassified | 7 | 9 | 4 | 4 | 21 | 16 | 17 | 21 | 25 | 9 | 9 | 10 |
| Microbacterium | 42 | 70 | 115 | 92 | 132 | 76 | 298 | 736 | 105 | 50 | 411 | 174 |
| Microcoleus | 15 | 42 | 131 | 19 | 50 | 81 | 11 | 20 | 28 | 78 | 142 | 47 |
| Microgenomates_norank | 15 | 22 | 5 | 11 | 22 | 25 | 13 | 8 | 17 | 34 | 14 | 6 |
| Microlunatus | 7 | 12 | 13 | 17 | 44 | 34 | 16 | 29 | 24 | 19 | 15 | 24 |
| Micromonosporaceae_Unclassified | 48 | 57 | 31 | 25 | 130 | 61 | 48 | 49 | 54 | 45 | 34 | 25 |
| Microvirga | 35 | 48 | 39 | 50 | 94 | 87 | 56 | 55 | 161 | 82 | 40 | 46 |
| Mitochondria_norank | 9529 | 5524 | 6646 | 8738 | 4941 | 1214 | 2319 | 1752 | 2903 | 3880 | 3717 | 5134 |
| Mizugakiibacter | 39 | 165 | 131 | 97 | 48 | 124 | 346 | 183 | 45 | 582 | 87 | 33 |
| Mobilitalea | 0 | 1 | 1 | 1 | 5 | 68 | 21 | 1 | 31 | 3 | 1 | 0 |
| Moraxellaceae_uncultured | 19 | 11 | 10 | 10 | 12 | 4 | 7 | 7 | 12 | 17 | 11 | 3 |
| Mucilaginibacter | 174 | 330 | 113 | 137 | 352 | 347 | 894 | 2282 | 268 | 484 | 607 | 80 |
| Mycobacterium | 12 | 28 | 27 | 30 | 51 | 53 | 62 | 69 | 96 | 53 | 52 | 40 |
| Myxococcaceae_Unclassified | 10 | 10 | 10 | 12 | 34 | 15 | 16 | 14 | 33 | 12 | 7 | 14 |
| Myxococcales_Unclassified | 17 | 30 | 21 | 39 | 63 | 52 | 33 | 36 | 49 | 41 | 18 | 16 |
| Myxococcales_uncultured | 13 | 19 | 10 | 10 | 35 | 23 | 9 | 5 | 26 | 18 | 3 | 14 |
| NKB5_norank | 19 | 11 | 23 | 19 | 61 | 35 | 18 | 14 | 18 | 16 | 6 | 3 |
| NPL-UPA2_norank | 2 | 8 | 6 | 2 | 6 | 5 | 3 | 0 | 12 | 6 | 3 | 0 |
| NS11-12 marine group_norank | 44 | 24 | 23 | 18 | 49 | 40 | 22 | 11 | 98 | 42 | 14 | 11 |
| NS9 marine group_norank | 6 | 11 | 8 | 3 | 6 | 15 | 4 | 2 | 10 | 8 | 3 | 3 |
| Nakamurella | 4 | 17 | 13 | 30 | 37 | 40 | 28 | 33 | 43 | 20 | 21 | 22 |
| Nannocystaceae_Unclassified | 3 | 5 | 4 | 2 | 13 | 3 | 3 | 7 | 6 | 2 | 2 | 2 |
| Nannocystaceae_uncultured | 21 | 5 | 5 | 12 | 22 | 49 | 12 | 25 | 18 | 19 | 10 | 1 |
| Nannocystis | 30 | 76 | 16 | 22 | 61 | 54 | 36 | 41 | 49 | 28 | 38 | 12 |
| Neisseriaceae_uncultured | 5 | 0 | 1 | 1 | 6 | 11 | 0 | 0 | 3 | 0 | 2 | 13 |
| Neptunomonas | 0 | 0 | 0 | 0 | 2 | 0 | 0 | 1 | 1 | 1 | 0 | 0 |
| Niabella | 3 | 2 | 3 | 0 | 3 | 7 | 18 | 22 | 3 | 7 | 4 | 1 |
| Niastella | 783 | 269 | 191 | 142 | 1091 | 369 | 239 | 242 | 873 | 451 | 386 | 185 |
| Nitratireductor | 31 | 62 | 56 | 103 | 104 | 107 | 171 | 244 | 66 | 87 | 71 | 61 |
| Nitrolancea | 0 | 1 | 0 | 1 | 0 | 2 | 10 | 14 | 0 | 1 | 0 | 0 |
| Nitrosomonadaceae_uncultured | 374 | 579 | 423 | 522 | 1004 | 832 | 467 | 366 | 967 | 650 | 295 | 293 |
| Nitrosomonadales_Unclassified | 1 | 2 | 0 | 5 | 2 | 1 | 1 | 1 | 4 | 5 | 0 | 2 |
| Nitrosomonas | 2 | 1 | 7 | 13 | 11 | 4 | 9 | 4 | 12 | 2 | 2 | 11 |
| Nitrosospira | 18 | 29 | 62 | 137 | 56 | 49 | 96 | 89 | 38 | 31 | 58 | 63 |
| Nitrospira | 107 | 210 | 211 | 281 | 457 | 326 | 209 | 223 | 324 | 210 | 217 | 139 |
| Nocardioides | 79 | 171 | 135 | 167 | 293 | 244 | 309 | 813 | 223 | 217 | 412 | 247 |
| Nonomuraea | 1 | 0 | 1 | 3 | 2 | 0 | 0 | 2 | 1 | 2 | 1 | 0 |
| Nordella | 93 | 183 | 93 | 127 | 261 | 210 | 151 | 159 | 224 | 140 | 105 | 71 |
| Nostoc | 5 | 4 | 2 | 5 | 5 | 5 | 8 | 9 | 56 | 4 | 15 | 3 |
| Noviherbaspirillum | 75 | 59 | 82 | 105 | 93 | 78 | 69 | 77 | 136 | 101 | 66 | 78 |
| Novosphingobium | 655 | 484 | 513 | 336 | 1216 | 1088 | 909 | 1886 | 1504 | 537 | 533 | 321 |
| OM190_norank | 23 | 29 | 14 | 23 | 64 | 49 | 17 | 18 | 48 | 38 | 21 | 8 |
| OM27 clade | 17 | 13 | 20 | 16 | 50 | 36 | 23 | 6 | 61 | 30 | 11 | 12 |
| OPB35 soil group_norank | 37 | 141 | 39 | 59 | 183 | 106 | 84 | 120 | 185 | 78 | 31 | 10 |
| OPB54_norank | 1 | 3 | 0 | 4 | 11 | 4 | 5 | 2 | 4 | 0 | 2 | 4 |
| OPB56_norank | 54 | 143 | 58 | 88 | 146 | 148 | 67 | 81 | 161 | 82 | 39 | 40 |
| Obscuribacterales_norank | 69 | 43 | 13 | 45 | 134 | 130 | 22 | 28 | 59 | 59 | 17 | 4 |
| Ochrobactrum | 8 | 6 | 3 | 3 | 8 | 11 | 18 | 45 | 9 | 9 | 35 | 6 |
| Ohtaekwangia | 334 | 345 | 217 | 234 | 478 | 367 | 188 | 130 | 413 | 319 | 198 | 134 |
| Oligoflexaceae_norank | 2 | 14 | 1 | 3 | 12 | 4 | 3 | 5 | 9 | 7 | 1 | 2 |
| Oligoflexales_norank | 80 | 50 | 36 | 40 | 259 | 89 | 74 | 75 | 154 | 57 | 35 | 13 |
| Oligoflexus | 7 | 13 | 5 | 11 | 16 | 12 | 10 | 6 | 27 | 3 | 8 | 4 |
| Olivibacter | 0 | 0 | 0 | 0 | 0 | 0 | 2 | 0 | 0 | 0 | 0 | 0 |
| Opitutae_Unclassified | 3 | 1 | 0 | 1 | 0 | 0 | 4 | 2 | 4 | 9 | 0 | 0 |
| Opitutus | 384 | 417 | 232 | 374 | 543 | 823 | 385 | 515 | 657 | 518 | 188 | 147 |
| Oryzihumus | 4 | 2 | 1 | 4 | 17 | 0 | 6 | 9 | 12 | 6 | 3 | 3 |
| Oscillatoria | 1 | 0 | 0 | 0 | 0 | 0 | 0 | 0 | 2 | 0 | 0 | 0 |
| Oscillochloris | 3 | 4 | 3 | 4 | 6 | 7 | 5 | 3 | 6 | 4 | 0 | 4 |
| Oxalobacteraceae_Unclassified | 149 | 195 | 208 | 495 | 160 | 170 | 233 | 160 | 234 | 326 | 206 | 185 |
| Oxalobacteraceae_uncultured | 10 | 6 | 8 | 5 | 11 | 14 | 24 | 8 | 10 | 11 | 10 | 10 |
| P2-11E_norank | 47 | 38 | 63 | 70 | 215 | 118 | 62 | 64 | 118 | 84 | 57 | 38 |
| P3OB-42_norank | 14 | 10 | 7 | 5 | 44 | 68 | 99 | 37 | 49 | 29 | 7 | 4 |
| PAUC26f_norank | 3 | 7 | 2 | 6 | 16 | 3 | 2 | 0 | 8 | 2 | 2 | 2 |
| PHOS-HE51_norank | 5 | 19 | 2 | 14 | 14 | 14 | 2 | 3 | 18 | 27 | 10 | 3 |
| Paenibacillaceae_Unclassified | 0 | 0 | 0 | 0 | 0 | 0 | 0 | 0 | 0 | 0 | 1 | 2 |
| Paenibacillus | 60 | 117 | 82 | 134 | 54 | 80 | 264 | 153 | 121 | 125 | 150 | 77 |
| Paludibacter | 0 | 0 | 0 | 1 | 2 | 4 | 3 | 0 | 112 | 0 | 0 | 0 |
| Panacagrimonas | 4 | 9 | 12 | 9 | 24 | 20 | 11 | 8 | 22 | 11 | 7 | 4 |
| Paracocccus | 0 | 2 | 3 | 1 | 3 | 9 | 4 | 6 | 59 | 3 | 8 | 2 |
| Parafilimonas | 4 | 13 | 3 | 11 | 19 | 24 | 16 | 9 | 9 | 14 | 8 | 3 |
| Parapedobacter | 0 | 0 | 0 | 0 | 0 | 6 | 7 | 2 | 0 | 0 | 0 | 0 |
| Parasegetibacter | 9 | 26 | 13 | 7 | 24 | 29 | 12 | 8 | 20 | 28 | 10 | 7 |
| Parcubacteria_norank | 6 | 8 | 7 | 8 | 10 | 21 | 9 | 10 | 9 | 13 | 3 | 4 |
| Parvibaculum | 0 | 0 | 0 | 2 | 0 | 1 | 3 | 0 | 1 | 0 | 0 | 0 |
| Patulibacter | 61 | 387 | 69 | 55 | 67 | 54 | 42 | 70 | 218 | 97 | 79 | 45 |
| PeM15_norank | 0 | 1 | 0 | 1 | 1 | 2 | 2 | 3 | 1 | 0 | 1 | 0 |
| Pectobacterium | 0 | 0 | 0 | 0 | 0 | 0 | 5 | 1 | 0 | 4 | 3 | 0 |
| Pedobacter | 21 | 12 | 17 | 14 | 15 | 16 | 51 | 40 | 50 | 71 | 39 | 8 |
| Pedomicrobium | 31 | 40 | 32 | 26 | 62 | 62 | 37 | 29 | 77 | 45 | 17 | 16 |
| Pelagibacterium | 1 | 3 | 1 | 1 | 5 | 6 | 0 | 12 | 1 | 6 | 6 | 0 |
| Pelomonas | 95 | 42 | 51 | 54 | 119 | 371 | 274 | 150 | 316 | 118 | 78 | 58 |
| Pelosinus | 0 | 1 | 4 | 0 | 8 | 10 | 12 | 0 | 13 | 1 | 4 | 3 |
| Peptococcaceae_Unclassified | 0 | 0 | 0 | 0 | 1 | 1 | 1 | 0 | 7 | 0 | 0 | 0 |
| Persicitalea | 0 | 0 | 0 | 0 | 0 | 6 | 3 | 7 | 2 | 0 | 3 | 0 |
| Phaeodactylibacter | 4 | 4 | 5 | 3 | 4 | 20 | 5 | 5 | 7 | 5 | 1 | 1 |
| Phaselicystis | 27 | 31 | 18 | 21 | 49 | 76 | 42 | 73 | 33 | 24 | 25 | 16 |
| Phenylobacterium | 499 | 335 | 334 | 349 | 763 | 718 | 724 | 1391 | 779 | 640 | 434 | 281 |
| Phormidium | 21 | 64 | 55 | 109 | 47 | 58 | 99 | 38 | 128 | 30 | 76 | 69 |
| Phreatobacter | 1 | 2 | 0 | 0 | 3 | 2 | 1 | 1 | 2 | 0 | 0 | 1 |
| Phycisphaeraceae_uncultured | 1 | 0 | 2 | 3 | 3 | 2 | 2 | 2 | 4 | 1 | 2 | 0 |
| Phycisphaerae_Unclassified | 0 | 1 | 0 | 0 | 0 | 0 | 0 | 1 | 0 | 0 | 1 | 0 |
| Phyllobacteriaceae_Unclassified | 16 | 19 | 20 | 15 | 34 | 39 | 40 | 51 | 27 | 19 | 27 | 10 |
| Phyllobacterium | 2 | 1 | 9 | 2 | 7 | 10 | 9 | 12 | 13 | 7 | 9 | 3 |
| Pir4 lineage | 11 | 23 | 5 | 10 | 24 | 16 | 10 | 14 | 37 | 9 | 10 | 5 |
| Pirellula | 66 | 146 | 82 | 132 | 188 | 176 | 104 | 131 | 191 | 116 | 79 | 58 |
| Piscinibacter | 1701 | 618 | 1057 | 1055 | 731 | 770 | 741 | 539 | 1198 | 555 | 452 | 352 |
| Pla3 lineage_norank | 0 | 0 | 1 | 1 | 2 | 0 | 1 | 0 | 0 | 2 | 0 | 1 |
| Pla4 lineage_norank | 6 | 23 | 9 | 13 | 33 | 21 | 8 | 11 | 22 | 11 | 12 | 7 |
| Planctomyces | 96 | 126 | 89 | 89 | 160 | 170 | 132 | 116 | 234 | 115 | 84 | 61 |
| Planctomycetaceae_Unclassified | 6 | 21 | 20 | 13 | 26 | 19 | 11 | 20 | 31 | 21 | 11 | 4 |
| Planctomycetaceae_uncultured | 37 | 72 | 50 | 62 | 102 | 116 | 88 | 95 | 118 | 77 | 49 | 42 |
| Planctomycetes_Unclassified | 0 | 0 | 1 | 1 | 2 | 0 | 0 | 0 | 5 | 0 | 2 | 0 |
| Planktothrix | 0 | 4 | 1 | 5 | 3 | 0 | 2 | 1 | 28 | 2 | 0 | 2 |
| Planomonospora | 0 | 1 | 1 | 1 | 2 | 2 | 1 | 2 | 1 | 1 | 0 | 1 |
| Pleomorphomonas | 34 | 34 | 27 | 26 | 110 | 286 | 112 | 37 | 117 | 74 | 10 | 9 |
| Polaromonas | 18 | 22 | 21 | 22 | 32 | 27 | 40 | 26 | 50 | 14 | 8 | 12 |
| Polyangiaceae_Unclassified | 11 | 26 | 16 | 17 | 41 | 39 | 19 | 19 | 26 | 20 | 16 | 10 |
| Polyangiaceae_uncultured | 15 | 54 | 26 | 24 | 57 | 84 | 38 | 39 | 26 | 50 | 23 | 12 |
| Polyangium | 33 | 21 | 16 | 17 | 57 | 35 | 28 | 15 | 43 | 38 | 8 | 8 |
| Polycyclovorans | 10 | 46 | 16 | 25 | 49 | 57 | 29 | 24 | 42 | 43 | 14 | 15 |
| Polynucleobacter | 3 | 16 | 3 | 7 | 5 | 3 | 5 | 13 | 3 | 3 | 4 | 1 |
| Promicromonospora | 1 | 2 | 2 | 0 | 0 | 0 | 0 | 3 | 0 | 2 | 1 | 0 |
| Propionibacteriaceae_Unclassified | 1 | 1 | 0 | 3 | 3 | 13 | 14 | 5 | 6 | 2 | 0 | 0 |
| Propionibacteriaceae_uncultured | 5 | 5 | 8 | 6 | 10 | 8 | 12 | 13 | 8 | 7 | 9 | 5 |
| Propionibacteriales_Unclassified | 0 | 2 | 1 | 3 | 4 | 2 | 4 | 4 | 3 | 3 | 6 | 5 |
| Propionibacterium | 0 | 1 | 0 | 1 | 2 | 0 | 0 | 0 | 4 | 1 | 2 | 1 |
| Propionivibrio | 0 | 0 | 0 | 0 | 0 | 0 | 2 | 0 | 7 | 2 | 0 | 0 |
| Prosthecobacter | 1 | 0 | 1 | 0 | 3 | 0 | 0 | 1 | 2 | 0 | 1 | 0 |
| Prosthecomicrobium | 4 | 3 | 8 | 4 | 16 | 22 | 17 | 8 | 26 | 5 | 3 | 5 |
| Proteobacteria_Unclassified | 21 | 19 | 27 | 33 | 112 | 67 | 32 | 43 | 64 | 27 | 19 | 13 |
| Pseudenhygromyxa | 14 | 11 | 9 | 12 | 30 | 17 | 6 | 1 | 30 | 7 | 4 | 6 |
| Pseudobacteroides | 0 | 0 | 0 | 2 | 4 | 2 | 5 | 0 | 17 | 0 | 1 | 0 |
| Pseudoduganella | 801 | 236 | 284 | 171 | 606 | 779 | 488 | 911 | 1110 | 732 | 442 | 283 |
| Pseudogulbenkiania | 0 | 0 | 0 | 0 | 4 | 0 | 0 | 5 | 2 | 2 | 0 | 0 |
| Pseudolabrys | 91 | 142 | 126 | 140 | 308 | 223 | 215 | 244 | 137 | 167 | 81 | 61 |
| Pseudomonas | 131 | 137 | 141 | 86 | 168 | 262 | 135 | 278 | 1005 | 246 | 141 | 92 |
| Pseudonocardia | 21 | 74 | 41 | 25 | 54 | 33 | 36 | 34 | 83 | 33 | 25 | 26 |
| Pseudonocardiaceae_Unclassified | 0 | 0 | 0 | 1 | 0 | 0 | 0 | 0 | 8 | 0 | 0 | 0 |
| Pseudospirillum | 2 | 4 | 2 | 0 | 3 | 2 | 5 | 1 | 8 | 6 | 4 | 3 |
| Pseudoxanthomonas | 19 | 38 | 15 | 32 | 147 | 344 | 145 | 265 | 108 | 125 | 80 | 39 |
| Pusillimonas | 1 | 1 | 1 | 9 | 1 | 2 | 14 | 24 | 0 | 0 | 9 | 19 |
| RB41_norank | 350 | 1156 | 429 | 605 | 1087 | 1299 | 653 | 633 | 1094 | 984 | 641 | 359 |
| RB446_norank | 1 | 0 | 2 | 2 | 1 | 6 | 2 | 0 | 6 | 2 | 1 | 1 |
| Ramlibacter | 289 | 304 | 296 | 402 | 510 | 803 | 469 | 457 | 816 | 408 | 199 | 204 |
| Reyranella | 75 | 121 | 90 | 99 | 173 | 166 | 122 | 107 | 170 | 103 | 62 | 70 |
| Rheinheimera | 59 | 12 | 9 | 36 | 2 | 6 | 2 | 3 | 27 | 40 | 16 | 13 |
| Rhizobiales_Unclassified | 23 | 31 | 28 | 21 | 76 | 28 | 25 | 33 | 55 | 36 | 15 | 12 |
| Rhizobiales_uncultured | 2 | 4 | 5 | 0 | 3 | 6 | 2 | 2 | 4 | 4 | 1 | 0 |
| Rhizobium | 583 | 482 | 285 | 346 | 703 | 863 | 550 | 1340 | 800 | 511 | 603 | 307 |
| Rhizomicrobium | 68 | 122 | 90 | 122 | 195 | 211 | 473 | 209 | 178 | 168 | 62 | 31 |
| Rhodanobacter | 119 | 496 | 821 | 1328 | 514 | 553 | 2372 | 2482 | 247 | 537 | 1501 | 844 |
| Rhodobacter | 37 | 49 | 43 | 35 | 33 | 45 | 14 | 26 | 69 | 37 | 21 | 44 |
| Rhodobacteraceae_uncultured | 0 | 0 | 1 | 0 | 1 | 2 | 0 | 0 | 0 | 0 | 0 | 0 |
| Rhodobiaceae_uncultured | 64 | 118 | 77 | 119 | 253 | 159 | 99 | 105 | 133 | 106 | 74 | 60 |
| Rhodococcus | 7 | 12 | 19 | 16 | 25 | 42 | 42 | 37 | 17 | 13 | 36 | 26 |
| Rhodocyclaceae_Unclassified | 38 | 13 | 27 | 62 | 257 | 590 | 361 | 46 | 666 | 72 | 13 | 4 |
| Rhodocyclaceae_uncultured | 0 | 0 | 0 | 0 | 0 | 0 | 7 | 4 | 41 | 0 | 0 | 0 |
| Rhodocytophaga | 2 | 7 | 1 | 2 | 3 | 8 | 3 | 2 | 14 | 4 | 4 | 2 |
| Rhodomicrobium | 3 | 4 | 0 | 1 | 25 | 4 | 2 | 2 | 5 | 4 | 3 | 1 |
| Rhodopirellula | 6 | 4 | 5 | 8 | 3 | 8 | 11 | 18 | 6 | 7 | 1 | 4 |
| Rhodoplanes | 37 | 66 | 55 | 38 | 115 | 115 | 37 | 65 | 93 | 66 | 39 | 38 |
| Rhodopseudomonas | 4 | 5 | 23 | 28 | 27 | 43 | 67 | 122 | 15 | 30 | 40 | 15 |
| Rhodospirillaceae_Unclassified | 16 | 3 | 5 | 3 | 20 | 3 | 3 | 2 | 12 | 5 | 1 | 3 |
| Rhodospirillaceae_uncultured | 81 | 87 | 65 | 62 | 177 | 184 | 87 | 109 | 140 | 106 | 55 | 56 |
| Rhodospirillales_Unclassified | 23 | 15 | 18 | 15 | 23 | 11 | 16 | 13 | 19 | 28 | 6 | 9 |
| Rhodovarius | 0 | 0 | 0 | 1 | 2 | 0 | 0 | 0 | 2 | 0 | 0 | 1 |
| Rhodovastum | 1 | 0 | 5 | 1 | 4 | 3 | 22 | 10 | 4 | 3 | 2 | 2 |
| Rickettsiales_Unclassified | 2 | 5 | 2 | 1 | 2 | 1 | 4 | 3 | 3 | 3 | 0 | 0 |
| Roseiflexus | 416 | 552 | 386 | 334 | 673 | 887 | 435 | 617 | 795 | 655 | 404 | 263 |
| Rubellimicrobium | 23 | 12 | 23 | 15 | 24 | 38 | 21 | 25 | 87 | 25 | 27 | 19 |
| Rubritepida | 2 | 0 | 3 | 0 | 0 | 0 | 0 | 1 | 2 | 0 | 0 | 0 |
| Rubrobacter | 0 | 1 | 0 | 1 | 1 | 0 | 0 | 1 | 2 | 1 | 2 | 0 |
| Rufibacter | 0 | 2 | 0 | 0 | 0 | 0 | 0 | 1 | 5 | 1 | 0 | 1 |
| Ruminococcaceae_Unclassified | 0 | 3 | 2 | 4 | 6 | 3 | 3 | 2 | 9 | 3 | 3 | 3 |
| Runella | 6 | 1 | 0 | 6 | 3 | 13 | 2 | 2 | 10 | 7 | 2 | 1 |
| S0134 terrestrial group_norank | 9 | 27 | 16 | 31 | 52 | 35 | 19 | 24 | 33 | 26 | 17 | 14 |
| S085_norank | 3 | 19 | 8 | 11 | 22 | 22 | 13 | 18 | 18 | 14 | 9 | 7 |
| S15-21_norank | 1 | 0 | 0 | 0 | 1 | 1 | 0 | 0 | 1 | 0 | 0 | 0 |
| SC-I-84_norank | 183 | 288 | 219 | 277 | 468 | 491 | 315 | 246 | 419 | 252 | 170 | 182 |
| SHA-109_norank | 3 | 4 | 6 | 6 | 24 | 23 | 13 | 14 | 21 | 12 | 4 | 5 |
| SHA-26_norank | 4 | 2 | 9 | 7 | 20 | 15 | 5 | 9 | 11 | 7 | 6 | 4 |
| SJA-149_norank | 1 | 6 | 0 | 4 | 9 | 98 | 28 | 8 | 9 | 6 | 2 | 1 |
| SJA-28_norank | 5 | 7 | 2 | 1 | 7 | 11 | 4 | 6 | 13 | 8 | 6 | 3 |
| SM1A02 | 5 | 2 | 3 | 1 | 11 | 15 | 4 | 4 | 9 | 9 | 1 | 4 |
| SM2D12_norank | 5 | 15 | 6 | 4 | 10 | 7 | 3 | 2 | 12 | 8 | 6 | 2 |
| SM2F11_norank | 19 | 21 | 12 | 11 | 15 | 28 | 21 | 14 | 25 | 38 | 16 | 5 |
| SPOTSOCT00m83_norank | 0 | 0 | 0 | 0 | 1 | 2 | 0 | 0 | 3 | 0 | 2 | 0 |
| Saccharibacteria_norank | 112 | 433 | 58 | 68 | 67 | 102 | 118 | 122 | 356 | 134 | 79 | 26 |
| Salinisphaeraceae_uncultured | 0 | 2 | 1 | 4 | 5 | 3 | 3 | 1 | 3 | 2 | 1 | 3 |
| Sandaracinaceae_norank | 3 | 2 | 0 | 0 | 4 | 3 | 2 | 2 | 0 | 3 | 1 | 0 |
| Sandaracinaceae_uncultured | 76 | 33 | 31 | 40 | 99 | 61 | 49 | 46 | 81 | 45 | 31 | 29 |
| Sandaracinus | 5 | 9 | 6 | 5 | 16 | 31 | 17 | 11 | 9 | 12 | 4 | 5 |
| Saprospiraceae_uncultured | 37 | 135 | 68 | 48 | 128 | 159 | 69 | 77 | 135 | 112 | 53 | 37 |
| Schlesneria | 9 | 14 | 11 | 11 | 17 | 41 | 23 | 21 | 24 | 13 | 17 | 4 |
| Scytonema | 0 | 0 | 0 | 0 | 0 | 0 | 0 | 0 | 4 | 0 | 0 | 0 |
| Segetibacter | 6 | 17 | 8 | 18 | 14 | 23 | 11 | 15 | 28 | 17 | 18 | 10 |
| Selenomonadales_uncultured | 1 | 4 | 3 | 10 | 12 | 81 | 13 | 7 | 96 | 9 | 3 | 1 |
| Sh765B-TzT-29_norank | 1 | 2 | 1 | 1 | 7 | 5 | 3 | 2 | 7 | 2 | 1 | 4 |
| Shinella | 323 | 396 | 329 | 457 | 352 | 417 | 367 | 619 | 577 | 287 | 262 | 212 |
| Simkaniaceae_uncultured | 0 | 0 | 0 | 0 | 2 | 1 | 1 | 0 | 0 | 0 | 0 | 0 |
| Singulisphaera | 3 | 7 | 6 | 12 | 14 | 19 | 34 | 18 | 23 | 11 | 9 | 10 |
| Skermanella | 27 | 30 | 47 | 45 | 126 | 76 | 57 | 45 | 186 | 52 | 49 | 48 |
| Smaragdicoccus | 2 | 8 | 1 | 3 | 5 | 12 | 13 | 11 | 5 | 5 | 7 | 10 |
| Sneathiellaceae_uncultured | 0 | 1 | 1 | 1 | 4 | 1 | 1 | 0 | 0 | 1 | 1 | 1 |
| Solirubrobacter | 30 | 35 | 51 | 48 | 84 | 91 | 44 | 73 | 66 | 53 | 38 | 35 |
| Solirubrobacterales_Unclassified | 4 | 15 | 12 | 13 | 27 | 30 | 18 | 21 | 17 | 15 | 12 | 24 |
| Solitalea | 16 | 12 | 17 | 8 | 8 | 8 | 11 | 4 | 57 | 6 | 6 | 1 |
| Sorangium | 26 | 42 | 14 | 21 | 56 | 81 | 37 | 57 | 58 | 38 | 27 | 22 |
| Sphingobacteriaceae_Unclassified | 53 | 96 | 40 | 54 | 37 | 73 | 39 | 73 | 83 | 62 | 51 | 48 |
| Sphingobacteriales_Unclassified | 0 | 0 | 0 | 0 | 3 | 2 | 0 | 3 | 2 | 0 | 1 | 0 |
| Sphingobacterium | 3 | 4 | 1 | 0 | 11 | 9 | 100 | 91 | 58 | 24 | 72 | 16 |
| Sphingobium | 141 | 65 | 35 | 35 | 660 | 605 | 224 | 437 | 342 | 61 | 163 | 23 |
| Sphingomonadaceae_Unclassified | 256 | 225 | 157 | 142 | 497 | 612 | 402 | 1263 | 261 | 394 | 200 | 169 |
| Sphingomonadales_Unclassified | 0 | 0 | 0 | 0 | 0 | 3 | 0 | 0 | 2 | 0 | 0 | 0 |
| Sphingomonadales_norank | 2 | 1 | 0 | 0 | 1 | 7 | 4 | 6 | 2 | 1 | 0 | 0 |
| Sphingomonas | 179 | 429 | 129 | 142 | 546 | 445 | 556 | 1141 | 563 | 981 | 344 | 140 |
| Sphingopyxis | 16 | 27 | 13 | 28 | 38 | 60 | 60 | 71 | 23 | 18 | 26 | 20 |
| Spirochaeta 2 | 15 | 0 | 2 | 1 | 33 | 2 | 10 | 0 | 30 | 4 | 6 | 1 |
| Spirochaetaceae_Unclassified | 0 | 0 | 0 | 0 | 9 | 1 | 1 | 0 | 0 | 2 | 0 | 0 |
| Sporichthya | 0 | 0 | 1 | 0 | 1 | 1 | 3 | 2 | 1 | 0 | 0 | 0 |
| Sporichthyaceae_Unclassified | 0 | 8 | 2 | 5 | 0 | 0 | 0 | 3 | 3 | 0 | 0 | 3 |
| Sporichthyaceae_norank | 6 | 22 | 6 | 16 | 1 | 0 | 0 | 1 | 5 | 7 | 5 | 2 |
| Sporichthyaceae_uncultured | 9 | 16 | 14 | 11 | 27 | 23 | 19 | 24 | 24 | 17 | 12 | 14 |
| Sporocytophaga | 24 | 6 | 10 | 17 | 65 | 29 | 12 | 3 | 25 | 43 | 4 | 4 |
| Sporomusa | 0 | 0 | 0 | 0 | 4 | 19 | 10 | 1 | 8 | 0 | 0 | 1 |
| Starkeya | 1 | 1 | 2 | 2 | 3 | 14 | 2 | 6 | 4 | 6 | 3 | 0 |
| Stenotrophomonas | 7 | 37 | 0 | 1 | 16 | 13 | 54 | 70 | 24 | 14 | 60 | 11 |
| Steroidobacter | 242 | 90 | 68 | 99 | 283 | 120 | 87 | 55 | 183 | 148 | 97 | 65 |
| Streptomyces | 1011 | 726 | 650 | 688 | 1222 | 433 | 581 | 973 | 942 | 796 | 854 | 618 |
| Streptosporangium | 1 | 4 | 1 | 2 | 11 | 10 | 5 | 13 | 9 | 14 | 12 | 6 |
| Subgroup 11_norank | 2 | 12 | 4 | 5 | 13 | 10 | 10 | 7 | 23 | 6 | 4 | 6 |
| Subgroup 12_norank | 2 | 11 | 9 | 7 | 32 | 17 | 10 | 5 | 10 | 8 | 6 | 2 |
| Subgroup 15_norank | 2 | 7 | 5 | 10 | 15 | 8 | 2 | 6 | 10 | 5 | 2 | 0 |
| Subgroup 17_norank | 37 | 45 | 40 | 29 | 120 | 94 | 58 | 48 | 108 | 95 | 33 | 40 |
| Subgroup 18_norank | 4 | 3 | 2 | 0 | 6 | 1 | 8 | 6 | 5 | 4 | 6 | 1 |
| Subgroup 25_norank | 14 | 24 | 11 | 21 | 44 | 33 | 28 | 25 | 31 | 26 | 15 | 9 |
| Subgroup 2_norank | 6 | 15 | 16 | 16 | 56 | 44 | 19 | 20 | 30 | 23 | 8 | 16 |
| Subgroup 3_Unclassified | 1 | 13 | 5 | 3 | 9 | 12 | 4 | 6 | 9 | 7 | 2 | 3 |
| Subgroup 5_norank | 12 | 45 | 34 | 25 | 89 | 53 | 33 | 35 | 63 | 57 | 35 | 21 |
| Subgroup 6_norank | 494 | 1074 | 762 | 896 | 2064 | 1456 | 737 | 806 | 1598 | 1063 | 683 | 512 |
| Subgroup 7_norank | 247 | 613 | 363 | 389 | 995 | 905 | 487 | 521 | 779 | 523 | 325 | 224 |
| SubsectionIII_FamilyI_Unclassified | 5 | 18 | 4 | 11 | 39 | 15 | 12 | 14 | 32 | 8 | 15 | 20 |
| SubsectionIII_FamilyI_norank | 0 | 1 | 7 | 0 | 1 | 1 | 0 | 0 | 33 | 8 | 2 | 0 |
| SubsectionIII_FamilyI_uncultured | 21 | 35 | 29 | 34 | 44 | 27 | 23 | 10 | 70 | 28 | 22 | 18 |
| SubsectionIV_FamilyI_norank | 19 | 23 | 18 | 10 | 191 | 61 | 65 | 33 | 68 | 95 | 34 | 46 |
| SubsectionI_FamilyI_norank | 2 | 0 | 2 | 96 | 2 | 0 | 0 | 0 | 0 | 0 | 0 | 0 |
| SubsectionI_FamilyI_uncultured | 3 | 13 | 21 | 17 | 13 | 6 | 3 | 4 | 17 | 1 | 2 | 1 |
| Sulfuricurvum | 0 | 1 | 0 | 1 | 2 | 1 | 9 | 0 | 112 | 0 | 0 | 0 |
| Sulfurospirillum | 0 | 0 | 0 | 0 | 0 | 2 | 0 | 0 | 21 | 0 | 0 | 0 |
| Sva0725_norank | 8 | 11 | 9 | 10 | 21 | 15 | 15 | 14 | 20 | 12 | 4 | 7 |
| Syntrophaceae_uncultured | 0 | 1 | 0 | 1 | 2 | 2 | 1 | 0 | 1 | 1 | 2 | 0 |
| TA18_norank | 3 | 2 | 0 | 1 | 2 | 2 | 1 | 0 | 1 | 1 | 1 | 2 |
| TK10_norank | 49 | 106 | 93 | 99 | 205 | 156 | 89 | 93 | 151 | 129 | 60 | 64 |
| TK34_norank | 0 | 6 | 6 | 6 | 21 | 13 | 5 | 6 | 22 | 10 | 0 | 2 |
| TM146_norank | 4 | 11 | 6 | 11 | 13 | 12 | 7 | 6 | 11 | 11 | 20 | 10 |
| TM6_norank | 23 | 16 | 19 | 22 | 18 | 13 | 7 | 10 | 24 | 13 | 7 | 1 |
| TRA3-20_norank | 56 | 105 | 65 | 80 | 161 | 181 | 82 | 76 | 152 | 101 | 63 | 36 |
| Tahibacter | 26 | 16 | 15 | 12 | 47 | 74 | 72 | 68 | 55 | 22 | 40 | 10 |
| Taibaiella | 6 | 3 | 1 | 0 | 8 | 16 | 23 | 18 | 31 | 23 | 25 | 11 |
| Telmatobacter | 6 | 33 | 39 | 26 | 44 | 44 | 54 | 41 | 2 | 67 | 19 | 3 |
| Terrabacter | 17 | 34 | 24 | 38 | 44 | 47 | 27 | 92 | 40 | 35 | 39 | 33 |
| Terriglobus | 6 | 12 | 2 | 2 | 4 | 7 | 6 | 12 | 0 | 15 | 15 | 3 |
| Terrimonas | 54 | 114 | 52 | 52 | 110 | 168 | 96 | 83 | 130 | 107 | 61 | 30 |
| Thalassospira | 1 | 0 | 0 | 2 | 9 | 1 | 1 | 0 | 0 | 1 | 1 | 0 |
| Thermincola | 0 | 0 | 1 | 1 | 1 | 3 | 0 | 0 | 1 | 1 | 0 | 1 |
| Thermomonas | 53 | 114 | 90 | 66 | 100 | 264 | 169 | 271 | 95 | 103 | 181 | 86 |
| Thiobacillus | 0 | 1 | 0 | 2 | 3 | 13 | 8 | 3 | 5 | 0 | 0 | 0 |
| Thiohalophilus | 0 | 0 | 0 | 0 | 0 | 0 | 0 | 0 | 2 | 0 | 0 | 0 |
| Treponema 2 | 0 | 0 | 0 | 0 | 0 | 0 | 0 | 0 | 12 | 0 | 0 | 0 |
| Truepera | 0 | 1 | 0 | 0 | 0 | 0 | 1 | 1 | 1 | 0 | 1 | 0 |
| Tsukamurella | 0 | 0 | 3 | 0 | 1 | 1 | 0 | 5 | 0 | 1 | 0 | 0 |
| Tumebacillus | 4 | 8 | 11 | 27 | 18 | 10 | 25 | 13 | 41 | 14 | 16 | 12 |
| Turneriella | 1 | 0 | 0 | 0 | 10 | 0 | 1 | 0 | 5 | 1 | 0 | 0 |
| Uliginosibacterium | 3 | 0 | 0 | 0 | 8 | 57 | 84 | 3 | 8 | 23 | 1 | 1 |
| VHS-B3-70_norank | 8 | 12 | 6 | 4 | 23 | 41 | 14 | 19 | 16 | 5 | 5 | 3 |
| Vampirovibrionales_norank | 101 | 118 | 95 | 152 | 109 | 89 | 69 | 76 | 118 | 107 | 43 | 51 |
| Variibacter | 110 | 136 | 114 | 143 | 293 | 209 | 130 | 161 | 264 | 131 | 96 | 69 |
| Variovorax | 207 | 151 | 137 | 106 | 214 | 358 | 293 | 388 | 555 | 181 | 211 | 134 |
| Veillonellaceae_uncultured | 2 | 2 | 0 | 2 | 10 | 10 | 12 | 1 | 30 | 3 | 0 | 0 |
| Virgisporangium | 5 | 15 | 4 | 16 | 25 | 7 | 15 | 8 | 13 | 13 | 12 | 9 |
| Vogesella | 18 | 9 | 33 | 17 | 24 | 44 | 35 | 41 | 118 | 38 | 9 | 29 |
| WCHB1-32_norank | 2 | 2 | 0 | 0 | 26 | 271 | 105 | 6 | 247 | 40 | 4 | 1 |
| WCHB1-60_norank | 34 | 113 | 29 | 29 | 3 | 15 | 7 | 5 | 70 | 37 | 29 | 3 |
| WCHB1-69_norank | 0 | 0 | 0 | 0 | 1 | 1 | 0 | 1 | 20 | 0 | 0 | 0 |
| WD2101 soil group_norank | 63 | 151 | 95 | 121 | 174 | 221 | 122 | 172 | 185 | 143 | 88 | 84 |
| WD272_norank | 1 | 14 | 5 | 1 | 8 | 20 | 22 | 24 | 2 | 17 | 3 | 0 |
| Woodsholea | 0 | 0 | 1 | 2 | 3 | 2 | 2 | 1 | 4 | 1 | 0 | 0 |
| Xanthobacter | 2 | 1 | 0 | 0 | 5 | 19 | 11 | 14 | 3 | 1 | 1 | 0 |
| Xanthobacteraceae_Unclassified | 15 | 13 | 14 | 18 | 37 | 30 | 19 | 11 | 22 | 19 | 15 | 12 |
| Xanthobacteraceae_uncultured | 0 | 3 | 2 | 2 | 3 | 1 | 2 | 2 | 4 | 2 | 5 | 1 |
| Xanthomonadaceae_Unclassified | 28 | 42 | 46 | 34 | 59 | 102 | 113 | 141 | 92 | 74 | 88 | 51 |
| Xanthomonadaceae_uncultured | 29 | 61 | 27 | 27 | 48 | 102 | 62 | 84 | 50 | 93 | 26 | 34 |
| Xanthomonadales Incertae Sedis_uncultured | 8 | 21 | 11 | 12 | 41 | 32 | 16 | 7 | 29 | 21 | 14 | 11 |
| Xanthomonadales_Unclassified | 0 | 1 | 1 | 2 | 0 | 1 | 1 | 1 | 2 | 1 | 0 | 0 |
| Xanthomonadales_uncultured | 19 | 34 | 31 | 39 | 77 | 36 | 23 | 12 | 50 | 27 | 18 | 13 |
| YNPFFP1_norank | 1 | 9 | 10 | 11 | 15 | 9 | 14 | 12 | 11 | 10 | 9 | 7 |
| Zavarzinella | 3 | 2 | 5 | 1 | 5 | 6 | 3 | 2 | 4 | 1 | 1 | 3 |
| Zoogloea | 1 | 4 | 3 | 20 | 248 | 205 | 388 | 68 | 796 | 183 | 12 | 2 |
| alphaI cluster_norank | 21 | 13 | 12 | 13 | 28 | 43 | 30 | 24 | 45 | 21 | 14 | 16 |
| bacteriap25_norank | 0 | 2 | 0 | 0 | 1 | 2 | 0 | 0 | 1 | 2 | 0 | 0 |
| cvE6_norank | 8 | 22 | 4 | 3 | 16 | 11 | 2 | 1 | 5 | 10 | 5 | 3 |
| env.OPS 17_norank | 216 | 78 | 32 | 38 | 303 | 136 | 121 | 111 | 267 | 95 | 60 | 23 |
| hgcI clade | 1 | 7 | 5 | 2 | 0 | 0 | 3 | 3 | 6 | 1 | 6 | 2 |
| mle1-27_norank | 8 | 19 | 9 | 2 | 26 | 63 | 34 | 39 | 16 | 25 | 8 | 1 |
| possible genus 04 | 5 | 8 | 9 | 11 | 11 | 16 | 6 | 9 | 13 | 4 | 7 | 3 |
| vadinHA49_norank | 11 | 20 | 5 | 5 | 27 | 22 | 31 | 9 | 21 | 15 | 4 | 4 |
